# Exploring the Representations of Individual Entities in the Brain Combining EEG and Distributional Semantics

**DOI:** 10.3389/frai.2022.796793

**Published:** 2022-02-23

**Authors:** Andrea Bruera, Massimo Poesio

**Affiliations:** Cognitive Science Research Group, School of Electronic Engineering and Computer Science, Queen Mary University of London, London, United Kingdom

**Keywords:** brain decoding, proper names, individual entities, distributional semantics, language models, EEG, categories

## Abstract

Semantic knowledge about individual entities (i.e., the referents of proper names such as *Jacinta Ardern*) is fine-grained, episodic, and strongly social in nature, when compared with knowledge about generic entities (the referents of common nouns such as *politician*). We investigate the semantic representations of individual entities in the brain; and for the first time we approach this question using both neural data, in the form of newly-acquired EEG data, and distributional models of word meaning, employing them to isolate semantic information regarding individual entities in the brain. We ran two sets of analyses. The first set of analyses is only concerned with the evoked responses to individual entities and their categories. We find that it is possible to classify them according to both their coarse and their fine-grained category at appropriate timepoints, but that it is hard to map representational information learned from individuals to their categories. In the second set of analyses, we learn to decode from evoked responses to distributional word vectors. These results indicate that such a mapping can be learnt successfully: this counts not only as a demonstration that representations of individuals can be discriminated in EEG responses, but also as a first brain-based validation of distributional semantic models as representations of individual entities. Finally, in-depth analyses of the decoder performance provide additional evidence that the referents of proper names and categories have little in common when it comes to their representation in the brain.

## 1. Introduction

As the idiom goes, people and places can be one of a kind—but could it be that our brains actually mean it? Thinking about Jacinda Ardern involves inevitably bringing to mind the information that she's a politician, aside from her face and her voice. But together with this may come to mind much more: other fellow politicians, other people she is often portrayed with, or places where she's often found at. This bundle of information encompasses disparate pieces of knowledge about the person herself, her kind (or category), other people belonging to the same kind, and other parts of the world which are associated with her. But how is this bundle structured, and how are the various bits wound together in the brain?

It has been shown in cognitive neuroscience that relevant differences exist between the processing of semantic information regarding unique, individual entities, which are entities indicated by proper names such as people and places, and that related to generic entities, such as referents of common nouns (Semenza and Zettin, 1989; Gorno-Tempini and Price, 2001; Semenza, 2006, 2009; Martins and Farrajota, 2007; Olson et al., 2013; Fairhall et al., 2014; Brédart, 2017; Schneider et al., 2018; Morton et al., 2021). This distinction reflects two ontological distinctions. The first one holds between instances and categories, with roots in philosophy (Lowe, 2003; Murez and Recanati, 2016), formal linguistics (Carlson and Pelletier, 1995) and cognitive psychology (Rosch, 1975; Kahneman et al., 1992; Leslie et al., 1998; Carey and Xu, 2001). Intuitively, instances are entities which are perceived as unique, whereas categories are classes of individuals, grouped together in order to distinguish them according to their class, and not to their individual identity (Klapper et al., 2017). This is the sense in which categories and categorization will be intended in this work. The second distinction is a further qualification of the status of instances. It starts from the observation that whereas certain instances are generally given a proper name (for example people, places, monuments, pets), others typically are not (for instance, instances of chairs, pans, windows). The availability of a proper name as a label, according to this theory, reflects cognitive and social constraints: only individual, unique entities which are sufficiently cognitively and socially salient can receive a proper name (Strawson, 1950; Kripke, 1972; Jeshion, 2009). This is the sense in which we refer to individual entities.

Semantics investigations in cognitive neuroscience have mostly focused on generic entities, and individual entities are usually treated as special cases: early neuroimaging studies found a strong involvement of the anterior temporal lobes (ATLs) when processing individual entities (Gorno-Tempini et al., 1998; Grabowski et al., 2001), but the ATLs have since clearly emerged as a hub for semantic processing in general (Ralph et al., 2017). This puts into question the existence of separate loci of processing for individual and generic entities, as processing of the referents of proper names may just require more (and more wide-spread) resources, because of their high specificity (Borghesani et al., 2019), and because of their social and emotional features (Olson et al., 2007, 2013). Research on individual entities has then focused on finding the neural correlates of a possible supramodal representation of individual entities, specific to this kind of entity (Fairhall et al., 2014; Schneider et al., 2018; Tsantani et al., 2019); or, restricting the analysis to people, on understanding timing and location of uni- and multi- modal processes such as face and voice recognition (Campanella and Belin, 2007; Anzellotti and Caramazza, 2017; Young et al., 2020); on trying to tease apart the processes related to social and general semantic cognition (Olson et al., 2013; Rice et al., 2018; Binney and Ramsey, 2020); on the structure of the representations of people, by comparing associative and categorical priming for faces and names (Schweinberger, 1996; Wiese and Schweinberger, 2008; Wiese, 2011). The results in the literature with respect to this last question are contradictory, as it remains unclear whether the categorical priming effect is weaker (Young et al., 1994; Barry et al., 1998; Carson and Mike Burton, 2001; Vladeanu et al., 2006; Bazzanella and Bouquet, 2011; Germain-Mondon et al., 2011) or on a par with associative priming (Darling and Valentine, 2005; Stone and Valentine, 2007; Stone, 2008; Wiese and Schweinberger, 2011). More in general, it remains open to debate whether categorical information plays a significant role in the structuring the semantic representations of individuals, or not; and if it does, to what extent (Turk et al., 2005).

In artificial intelligence, and in particular in computational linguistics and NLP, recent advances have provided researchers with models, based on distributional properties of words in texts, which capture very subtle semantic knowledge (Erk, 2012; Camacho-Collados and Pilehvar, 2018). In particular, knowledge about individual entities can be now modeled in much greater detail and with impressive results in NLP benchmarks (Lenci et al., 2021). These representations of individual entities, however, have mostly been tested on traditional NLP tasks (Chen et al., 2019). Unlike with generic concepts (Mandera et al., 2017), very little attention has been paid to how well these models capture human processing of the semantics of individual entities. Distributional models of individual entities could, on the one hand, benefit from cognitive neuroscience research, which could provide a guide and an evaluation for the models (Günther et al., 2019; Hollenstein et al., 2019); and on the other, help it, by offering powerful vectorial models which can isolate processing of individual entities in the brain (Bruffaerts et al., 2019).

In this work we bring together brain data (in the form of electroencephalography, or EEG, data) and computational, distributional models of semantics. From the point of view of neuroscience, we investigate the way in which entity-exclusive and categorical (relating the entity to its category) semantic knowledge structures the semantic representations of both individual entities and their categories. From the point of view of computational linguistics, we look for the first time at whether distributional models encode semantic information about individual entities as it is processed in the brain.

In order to do this, we collected EEG data from 33 participants using linguistic stimuli. During the experiment we recorded responses to proper names of famous people and places, belonging to different categories (e.g., “Scarlett Johansson,” “Eiffel Tower”), as well as to the common nouns referring to their socially-relevant classes (e.g., “actor,” “monument”). This matched set of stimuli allowed us to investigate four questions: whether we could find distinctive signatures for each individual entity in the brain; to what extent these representations were shaped according to category; whether the evoked responses to individual entities and categories shared representational information, as picked up by machine learning models; and finally, to what extent distributional models map onto brain processing of individual entities.

We approach our research questions in two ways: one based on classification of EEG responses according to their category, both at a coarse- and fine-grained level of categorization; and another one based on learning regression models that perform decoding from brain data to word vectors (learning a linear map from brain activations to the true values of the word vectors' dimensions). We show that it is possible to obtain above-chance performance in both cases, finding traces of individualization and categorization, overcoming the high noise present in the EEG data. This also confirms that distributional models of individual entities, despite relying exclusively on textual data, encapsulate information matching brain processing. Furthermore, results indicate that evoked responses to nouns for categories and proper names for their instances have little representational information in common.

## 2. Background

### 2.1. Distributional Semantics

Distributional models of semantics represent the meaning of words in the form of vectors, by looking at co-occurrences between words as they are found in large collections of texts, called corpora (Lenci, 2008; Boleda, 2020).

The theoretical underpinning of these models dates back to Wittgenstein's considerations on language use (Wittgenstein, 1953). In his view, an important part of a word's meaning can be understood by looking at the way in which it is used in actual language. This so-called distributional hypothesis is best-known in the version formulated by Firth (1957): “You shall know a word by the company it keeps”–i.e., words which are found in similar contexts have similar meaning (see also Harris, 1954).

Vector-space models of lexical meaning based on the distributional hypothesis started to appear, and to be evaluated as cognitive models of lexical semantics, little more than 20 years ago, when both computational power and the availability of textual resources improved dramatically (Lund and Burgess, 1996; Landauer and Dumais, 1997; Schütze, 1997). The success of these models in, e.g., predicting human synonymy patterns (Turney, 2001) motivated a great deal of research (Lin, 1998; Finkelstein et al., 2001; Curran and Moens, 2002; Almuhareb and Poesio, 2004; Agirre and Edmonds, 2007; Bullinaria and Levy, 2007; Padó and Lapata, 2007; Baroni and Lenci, 2010).

After a first period in which the dominant paradigm was to learn such models out of word co-occurrences, the field moved toward deriving distributional models of words as by-products of neural networks whose objective was to learn language models (Mikolov et al., 2013; Baroni et al., 2014; Pennington et al., 2014), which is currently the dominant paradigm. These models frame word vector learning as a machine learning problem. The goal is that of learning, by looking at corpora, to predict word vectors such that words with similar meanings should have similar vectors, and vice versa. The latest models, which use deep neural networks (Peters et al., 2018; Devlin et al., 2019), take into account the fact word meaning changes slightly depending on the context where a word is found. In these models, there are no “static” word vectors; instead, they provide, given a sentence or a paragraph, word vectors specific to that context—and because of this, they are often called “contextualized” language models.

All along, it has been shown that these models capture semantic information about words as it is stored and processed in human cognition (Bruffaerts et al., 2019). This is quite surprising, given that these models rely exclusively on textual data, whereas humans have much broader sources of information—for recent overviews of what phenomena can be modeled, see Günther et al. (2019) and Lenci et al. (2021). In particular, research in cognitive neuroscience has shown the important role of sensory information in brain semantic representations (Barsalou, 2008; Ralph et al., 2017). In order to account for this type of signal, which cannot be found directly in text, another kind of distributional semantics models has been developed, adding also visual (Bruni et al., 2012) and auditory (Kiela and Clark, 2015) features to the vectors created from corpora. These multimodal distributional semantics models can, as expected, improve results on tasks involving concrete concepts (Bruni et al., 2014).

### 2.2. Cognitive Data and Distributional Semantics

Much early work on distributional models was evaluated in a purely qualitative matter, by showing that the lexical vectors clustered “in an intuitive way.” This informal sort of evaluation however was quickly replaced by attempts to introduce more quantitative forms of evaluation. One popular approach was to extract from a lexical database such as WordNet (Fellbaum, 1998) test words belonging to different classes (e.g., animals, tools) and then evaluate the extent to which the learned vectors clustered into clusters matching the original classes (Lin, 1998; Curran and Moens, 2002; Almuhareb and Poesio, 2004). However, this approach to evaluation made the results entirely depend on the cognitive plausibility of the classes in the target lexical database, which was problematic. Thus, while the knowledge-based approach was not completelely abandoned, it was quickly supplemented with forms of evaluation which tested more directly the extent to which the learned representations encoded linguistic and conceptual knowledge.

One type of approach is to use distributional models to predict human behavior in tests such as the already mentioned TOEFL test (Turney, 2001). In fact, this is the form of evaluation most used in the psychological literature from which distributional models originated (Lund and Burgess, 1996; Landauer and Dumais, 1997). Benchmarks for distributional models combining clustering evaluation with tests were proposed, e.g., by Baroni et al. (2010, 2014), and Lenci et al. (2021).

Another evaluation framework is that of similarity and relatedness tasks: given a set of words (for instance, concrete or abstract nouns), measures of similarity or relatedness between all possible pairs of words in the list are first elicited from human subjects (as simple examples, “cheese” and “yogurt” are very similar, whereas “cheese” and “France” are strongly related) and, in parallel, from a word vector model. For humans, some quantitative scale is used, whereas, for distributional models, a vector distance measure such as cosine similarity among word vectors is employed. Finally, the correlation between human judgments and the model's matched predictions is computed—the assumption being that the higher the distributional model's correlation with human ratings, the higher its quality (Finkelstein et al., 2001; Bruni et al., 2012; Hill et al., 2015). Yet another method is based on quantifying the models' ability to replicate cognitive phenomena such as priming and reading times (Jones and Mewhort, 2007; Mandera et al., 2017; Günther et al., 2019).

An exciting alternative to this approach was evaluating distributional models using evidence about semantic categorization in the brain (Warrington and Shallice, 1984; Caramazza and Shelton, 1998; Haxby et al., 2001). One of the earliest proposals exploring both brain data and distributional models of word meaning was Mitchell et al. (2008). That paper showed that, for a selected set of common nouns referring to concrete concepts, co-occurrence patterns as extracted from corpora could successfully predict brain activity evoked by drawings of the referent of the words and recorded with fMRI. fMRI stands for functional magnetic resonance imaging, and is a technique which provides images of neuronal activity as reflected by cerebral blood flow (Friston et al., 1998).

The approach of Mitchell et al. (2008), called brain encoding, was interesting for a number of reasons. First of all, it indicated that purely textual co-occurrence captured important features of semantic processing in the brain. Secondly, it pioneered an approach to isolate brain signatures of semantic processing of individual words using computational linguistics models—essentially turning what could be seen as a classification, categorical problem (recognizing the word which evoked the brain activity) to a regression problem (finding a mapping between one vectorial representation, previously obtained by way of a model, to another, the brain data). Finally, this work presented a new way of evaluating vectorial models of word meaning as created in computational linguistics, looking at how well they modeled brain processing. This effectively provided one of the most direct possible evaluations of the cognitive plausibility of the models.

After, Mitchell et al. (2008), several lines of work expanded the approach further. One approach focused on using different models of semantics on the same set of fMRI images provided by Mitchell et al. (2008). The goal was to try to find if, and how, performance differed across models: distributional models other than the ones originally used (Murphy et al., 2012), models incorporating knowledge base information (Jelodar et al., 2010), models based on Wikipedia definitions (Pereira et al., 2013), multimodal models incorporating both textual and visual features (Anderson et al., 2013), models based on word associations from a thesaurus (Akama et al., 2015). Another line of research involved obtaining original fMRI data, applying a similar encoding analysis, but widening the scope of the approach to other linguistic phenomena: phrases (Chang et al., 2009), sentences (Anderson et al., 2021), naturalistic processing of visually (Wehbe et al., 2014a) and orally (Huth et al., 2016; Zhang et al., 2020) presented stories.

A mirror approach was also proposed, that of decoding: the mapping is learnt from the fMRI brain data to the word vectors. In Anderson et al. (2017) the authors showed that not only representations of concrete concepts, but also abstract ones, could be successfully decoded to distributional word vectors. And in Pereira et al. (2018) a much more extensive set of stimuli was used, encompassing both abstract and concrete concepts, presented as words, definitional sentences and pictures during the fMRI scans. Expanding even further the use of word vectors to isolate semantic processing in the brain, Djokic et al. (2020) found evidence, through decoding to word vectors, that metaphorical and literal readings of sentences have different brain signatures, and Nishida and Nishimoto (2018) showed that it was possible to learn a mapping from videos (and their matched descriptive textual annotations) to distributional word vectors.

### 2.3. EEG and Distributional Semantics

Another type of brain data, whose main members are electroencephalography (EEG) and magnetoencephalography (MEG), has also been used for brain encoding/decoding studies from/to computational word vectors. They measure different signals coming from the brain (electrical fields for EEG, and magnetic fields for MEG), but their source is the same—ionic currents generated by biochemical processes in neurons (da Silva, 2013).

Although research on using EEG to evaluate distributional models started at about the same time as research using fMRI (Murphy et al., 2008, 2011), they have been used to a lesser extent, because of both conceptual and practical issues. fMRI captures extremely detailed brain images every 1 or 2 seconds. These are the ideal form of concept representation as retrieved from semantic memory, and can be straightforwardly matched to word vectors, which are taken to model lexical items in semantic memory. Instead, M/EEG provide snapshots of brain processing which have much better time resolution—in the milliseconds range—but poorer spatial definition. This is especially true in the case of EEG, which not only has the lowest spatial resolution of all, but also the lowest signal-to-noise ratio. Conceptually, word vectors do not model the temporal dimension of semantic processing, but rather the spatial one, in the form of distributed vector spaces: and spatial analyses in cognitive neuroscience research have traditionally involved primarily fMRI, due to its high spatial resolution (Kemmerer, 2014).

Both EEG and MEG come with their own advantages, however. EEG is particularly cheap and portable, two reasons which make it also the preferred choice for brain-computer interfaces (Allison et al., 2020) and for working with elderly patients (Gu et al., 2013); and, provided one uses enough channels, it can be used as a sort of brain imaging tool (Michel and Murray, 2012). MEG, despite being expensive, provides much better signal and spatial resolution, and more channels by default.

The few studies using EEG data implemented the encoding setup, as in Murphy et al. (2009), where pictures of concrete entities where used as stimuli; and both encoding and decoding in Sassenhagen and Fiebach (2020), a study where both concrete and abstract common nouns were used. MEG data, which grants higher machine learning performances given the superior signal quality, was instead used for decoding to word vectors from brain processing of pictures referring to concrete concepts (Sudre et al., 2012) and visually presented stories (Wehbe et al., 2014b).

With respect to the literature here reported, the main innovation of our work is showing that word vectors can be used for isolating extremely fine-grained brain signatures of individual entities indicated by proper names. By doing so, we go beyond knowledge about generic concepts indicated by common nouns, which was instead the main focus of the works reported above. Also, we show that it is possible to do so using the available technique with the lowest signal-to-noise ratio, EEG, provided that the experimental design is carefully designed.

## 3. Materials and Methods

### 3.1. Stimuli

**Figure 1:**
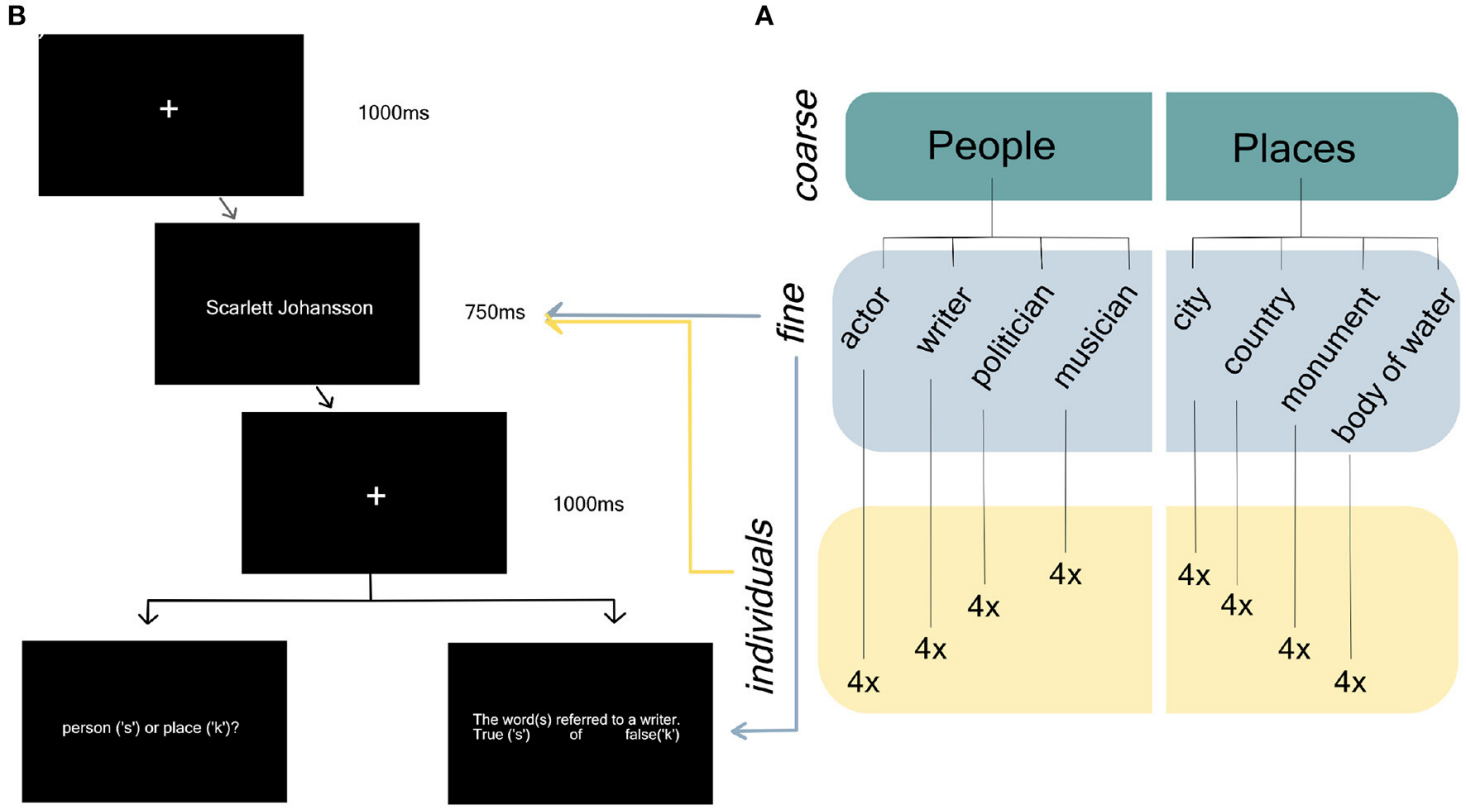
Experimental design and hierarchical stimuli organization. Our set of stimuli was organized symmetrically, so that exactly the same number of stimuli was present for each coarse- and fine-grained category—as shown in **(A)**. Stimuli included both proper names of individual entities and their fine-grained categories. In **(B)** we present the experimental paradigm. Each stimulus was projected in isolation on the screen for 750 ms, and subjects then had to mentally visualize its referent while a cross remained on screen for 1,000 ms. Participants then answered a question, involving the fine- or coarse-grained level of categorization.

In order to be able to investigate the structural properties of the representation of individual entities from both an entity-level and a categorical perspective, we collected evoked responses not only to proper names of people and places, but also to words referring to their main categories. Importantly, the set of stimuli is hierarchically structured, and carefully matched in terms of semantic categories and entities. There are two “coarse” categories (people and places); for each of these we considered four fine-grained categories (musicians, politicians, actors, writers as people, and bodies of water, monuments, cities and countries as places); and for each fine-grained category we included four individuals among the stimuli, as well as the nouns for the categories. The result is a total of 40 stimuli: 8 nouns for the fine-grained categories, and 32 proper names for the individual entities. The hierarchical structure of the stimuli and the way they are used in the experimental paradigm (Section 3.2) are presented in Figure 1, and the stimuli selection procedure is described below.

People and places were chosen a priori as top categories, following previous work on individual entities in cognitive neuroscience (Gorno-Tempini and Price, 2001; Grabowski et al., 2001; Ross and Olson, 2012; Fairhall and Caramazza, 2013; Leonardelli et al., 2019). In this field, people and places are the most common choice of categories to be contrasted when investigating the semantics of individual entities, since a long tradition of studies on patients has shown that, within human semantic knowledge of individual entities, these two categories can be selectively impaired (della Rocchetta et al., 1998; Miceli et al., 2000; McCarthy and Warrington, 2016). This seems to entail that semantic knowledge about these two kinds of individual entities can be teased apart in the brain—a finding which has consistently been confirmed by results from healthy subjects (Fairhall et al., 2014; Rice et al., 2018).

It should be noticed that in NLP, where textual resources can be obtained or created on a very large scale with limited effort, it is commonplace to consider a wider range of coarse-grained categories of entities (organizations, dates, events, products, or categories of biomedical entities such as genes and diseases Goyal et al., 2018). However, in cognitive neuroscience, it is much harder to obtain large scale datasets. This is due to the combination of two factors. The first one is practical: experimental sessions are particularly intense for subjects, imposing strong constraints on the number of trials that can be obtained and analyzed. The second one is methodological: in most cases, like ours, analyses are conducted at the level of individual subjects, and then aggregated at a later stage. This entails that the amount of experimental items that can be analyzed in the context of an experiment is limited to the number that can be considered for a single participant. The role of having multiple participants, instead, is not that of increasing the number of experimental items or trials, but that of testing the generalizability of the results.

For the fine-grained categories and the individuals, we followed a two-step, mixed approach, guiding our manual selection with data-driven observations. The aim of the first selection step was providing an initial, large set of individual entities and matched categories, to be further reduced during the second phase, using familiarity ratings provided by independent subjects.

For the first step, we came up with a list of ten fine-grained categories for people and places. We followed principles of economy and discriminativeness (Rosch, 1975), selecting, as fine-grained category stimuli, categories for people and places which represented economic ways of describing and distinguishing subclasses of individual entities. In the case of people, we used occupations as fine-grained categories (e.g., “politician” for individual concept “*Barack Obama*”), which is one of most basic, socially shared and clear-cut way of categorizing people (Cantor and Mischel, 1979; Mason and Macrae, 2004; Turk et al., 2005), whereas in the case of places we employed their taxonomic hypernym (e.g., “city” for individual entities such as “*Istanbul*”). The final list of ten categories included the eight categories reported above (musician, politician, actor, writer as people, and body of water, monument, city and country as places), as well as two additional entries, athlete and geographic area. Then, from this list of fine-grained categories, we manually picked a preliminary set of 100 well-known individual entities—10 names for each of 10 fine-grained categories. We made sure that each individual entity had a dedicated page in both English and Italian Wikipedia, since we needed that source of textual data in both languages for the extraction of word vectors, as discussed in Section 3.5.

For the second, and final, stimuli selection step, we chose familiarity as the main criterion, as it is one of the most important variables affecting the processing of proper names (Valentine, 1998; Smith-Spark et al., 2006; Brédart, 2017; Moore and Valentine, 2020), as well as a necessary requirement for our experiment (in order to capture the neural representations for an individual entity, we needed to ensure that subjects had a previous representation in semantic memory to be retrieved). Familiarity was defined, following Moore and Valentine (2020) as cumulative encounters with (representations of) that individual entity, across time and media. At the beginning of the second stimuli selection step, we collected familiarity ratings from 30 Italian subjects, none of which took part to the subsequent EEG experiment. We made sure that this sample was matched in nationality and close in age to the sample of the EEG experiment (mean age: 29), in order to ensure that the entities and the fine-grained categories used as stimuli for the data acquisition procedure would be as familiar as possible to our sample of participants in the EEG study. Note that this procedure was fully independent from EEG data collection: participants in the EEG experiment had no role in the stimuli selection procedure, and they were not asked to norm the stimuli according to familiarity. In the norming experiments, subjects were asked to rate on a Likert-type scale, from 1 to 5, their familiarity with each individual entity, where familiarity was defined as reported above.

After having collected the familiarity ratings, we retained, for the EEG study, only the individual entities and the fine-grained categories which, on average, had the highest familiarity scores. At the end of this procedure, the four most familiar fine-grained categories for each coarse category were chosen as fine-grained categories for the study; and the four most familiar individual entities belonging to those categories where selected as the individuals entities to be used. We report the set of stimuli in the Supplementary Materials.

Psycholinguistic variables such as orthographic complexity and length are particularly hard to match for proper names, especially in the case of our experimental setup. First of all, in languages such as Italian and English, they are morphologically and orthographically immediately recognizable from common names, because of features such as upper case initial letter, and non-applicability of number and case information (Peressotti et al., 2003). Secondly, proper names of people are in most cases longer than places' names, because they require both name and surname in order to be correctly disambiguated. Thirdly, there is no hope of matching proper names and words of fine-grained categories. And finally, since we use a decoding analysis centered around semantics, we assume that the decoders will automatically learn to focus on ERP signatures of semantic, not orthographic, information. Because of these reasons, we chose not to control rigidly orthographic variables, and in particular stimulus length, during data collection, taking for granted that such differences between stimuli would inevitably be present. Instead, we added word length as a control variable in the classification analyses (Chyzhyk et al., 2018), where it could play in principle a confounding role due to the differences in mean lengths across categories—9 letters for places, 12 letters for people (see Section 3.4 for the details, and the tables in the Supplementary Materials for the full list of the experimental stimuli, together with their familiarity scores and average word lengths).

### 3.2. Experimental Procedure

Thirty-tree right-handed subjects (age from 18 to 35 years old, with 20 female participants) took part to the experiment. The subjects were all native Italian speakers, and the experiment was conducted in Italian. All experimental procedures were approved by the Ethical Committee of SISSA, Trieste, where the data were collected, and subjects gave their written informed consent.

The experimental procedure involved 24 short runs of 40 trials. In each run, each of the 40 stimuli (both proper names and fine-grained category nouns) would appear once, in a randomized order, resulting, at the end of the session, in 24 evoked responses to each name and noun. We chose to repeat the stimuli 24 times, since (Grootswagers et al., 2017) clearly demonstrate that, in order to reach optimal decoding and classification results using evoked potentials, between 16 and 32 trials for each stimulus are needed. Each trial consisted of two parts, as shown in Figure 1: first, the presentation of a stimulus name or noun for 750 ms (word presentation times in EEG experiments are kept below 1 s, as word processing begins already at 150 ms after the stimulus appears Simanova et al., 2010; Wehbe et al., 2014b; Sassenhagen and Fiebach, 2020); the stimulus was preceded and followed by the presentation of a white fixation cross for 1,000 ms at the center of the screen. Participants were instructed to read the word and then visualize mentally the referent of the stimulus, until the cross was on screen. We decided to leave a relatively short time for the mental imagery task, in order to keep the process as much as possible time-locked to stimulus appearance (Bastiaansen et al., 2011; Shatek et al., 2019) and avoid mind wandering, while allowing subjects to quickly picture the stimulus' referent (cfr. Section 5.3).

Afterwards, a question appeared on screen, which always involved pressing either “s” or “k” on an external keyboard, placed in front of the subject. “s” and “k” were chosen simply because of their position on the keyboard's QWERTY layout: they can be easily reached with left and right index finger, respectively, while keeping a comfortable, yet fixed, position on the chair. There were two possible types of questions, either a coarse-level question (“people or place?”) or fine-level (e.g., “the name referred to a musician,” with possible answers “correct” or “wrong”), similarly to Leonardelli et al. (2019). The mapping between response keys and answers was fully randomized across trials. Within each run, questions were randomized, but balanced across question type and, when applicable, answer type: coarse-level questions appeared 16 times, whereas fine-level questions appeared 24 times, of which 12 times required a “correct” answer. The questions were added to the experimental paradigm in order to keep participants attentive and to ensure that they focused on their semantic representation of the individual entity and its category. The aim of randomizing different questions was avoiding as much as possible strategic preparation for the coming question.

### 3.3. EEG Recording and Preprocessing

For data acquisition, we employed a BIOSEMI ActiveTwo system with 128 channels ^1^, recording signals at a sampling rate of 2048 hz. We employed a fully automatized preprocessing pipeline adapted from Jas et al. (2018), and implemented with the MNE Python package (Gramfort et al., 2013), in order to improve replicability. Results were then visually inspected in order to check for the quality of the preprocessing, and we found that no amendment was required.

We first downsampled the data to 256 hz, a recommended choice for cognitive neuroscience experiments (Luck, 2014), leading to a sampling resolution of 3.9 ms. This reduces the amount of data points to be processed, speeding up the analyses, at no cost: as pointed out in Luck (2014), relevant cognitive activity happens at frequencies below 80 hz, and therefore, following Nyquist's theorem, sampling at 256hz is a safe choice (Nyquist's theorem states that, in order to capture in a digital format an analog signal, the sampling rate has to be more than twice as great as the highest frequency in the signal, here around 80 hz). Given the considerations above, we subsequently applied a low-pass filter to the data at 80hz so as to minimize the effect of irrelevant signal interferences.

A further source of noise in EEG recordings are voltage artifacts that have nothing to do with cognitive activity, such as slow voltage drifts. These are due to skin-related potentials, that generate noise in the recorded waveform because they have a shifted phase with respect to the evoked responses to the experimental stimuli (Luck, 2014). To remove them, we applied baseline correction, which consists in subtracting the average of the pre-stimulus potentials, in our case from –100 to 0 ms, from the whole epoch. This is the recommended alternative to the conceptually simpler high-pass filtering, which may instead induce undesired artifacts (Tanner et al., 2016; van Driel et al., 2021).

Finally, we epoched the data to –0.100 and 1,200 ms after stimulus onset, and used the AutoReject algorithm to find and interpolate bad channels and to remove bad epochs, excluding from the analyses spans of recorded data that contained excessive noise or artifacts (Jas et al., 2017).

We also recorded two electrooculogram (EOG) channels, which record electrical potentials produced by eye movements. These eye-related potentials, which may be picked up in parallel by EEG electrodes, interfere with the signal of interest coming from the brain, and are therefore considered as artifacts to be removed. The most common way of dealing with this kind of noise employs the recorded EOG channels together with Independent Component Analysis (ICA) (Urigüen and Garcia-Zapirain, 2015). ICA is used to separate linearly mixed signals, estimating their independent source signals from recorded data. In our case, we take the EEG recordings to contain a mixture of eye- and brain- related signals, that we would like to disentangle. In order to remove ocular artifacts, we used the standard procedure of Jas et al. (2018). Using the MNE implementation, we fit ICA on our data, then we found and excluded automatically the components which correlated the most with the EOG signal (Jas et al., 2018), the assumption being that these components capture eye-related signal sources.

To reduce the impact of noise, which can be quite severe in EEG recordings, we averaged all evoked responses corresponding to a stimulus within a subject, as it has been shown to improve the signal-to-noise ratio (Grootswagers et al., 2017). Before the analysis, we standardized the data for each channel using MNE's scaling method, which standardizes electric potentials channel by channel. For the standardization, mean and standard deviation are computed for each channel from all time points within all epochs.

This preprocessing pipeline provided us with 40 evoked responses for each stimulus per subject. Conceptually, these correspond to snapshots of semantic processing across 1,200 ms for the thirty-two proper names and their eight fine-grained categories.

### 3.4. Coarse- and Fine-Grained Decoding

Given the structure of our dataset, and our interest in uncovering the structure of semantic representations of individual entities at different levels of granularity, we carried out two separate classification analyses for the coarse-grained level (people and places) and the fine-grained level (the eight fine-grained categories). For both setups we employed a SVM with default parameters (C=1.0, *l*2 regularization). This is standard procedure in cognitive neuroscience, and it has been shown that, with *l*2-regularized SVM, fine-tuning the C parameter does not impact results (Grootswagers et al., 2017; Varoquaux et al., 2017). The main difference between coarse- and the fine-grained setups was that, whereas in the former case we set up a binary classification procedure, with random, baseline accuracy at 50%, in the latter we used multiclass, one-vs-all classification with random baseline at 12.5%. We will also present results for within-coarse categories (i.e., results obtained when using only evoked responses to either people or places). In this case too we used a one-vs-all classifier, this time with a baseline of 25%, since there are only four fine-grained categories to consider.

We took a time-resolved decoding approach (Grootswagers et al., 2017) for our classification, training and testing separately on each time point. We tested statistical significance at each time point using threshold-free cluster enhancement (TFCE), a permutation-based, non-parametric test of statistical significance proposed in Smith and Nichols (2009) and then widely adopted in the neuroscientific literature (Helwig, 2019), also for classification of EEG signals (Grootswagers et al., 2019; Kaiser et al., 2020; Petit et al., 2020). The main advantages of this procedure are its sensitivity, due to the fact that it can take into account the fact that brain signals are clustered both in space and in time; its avoidance of parametric test assumptions (Mensen and Khatami, 2013); and finally, the fact that it inherently counters the multiple comparisons problem (the inflated risk of finding false positives) that arises from testing so many data points (in our case, time points; for details on the general procedure, see Mensen and Khatami, 2013). We used the TFCE implementation of MNE (Gramfort et al., 2013), with default parameters. Since our classification took place in the time domain (time-point by time-point), the TFCE procedure could take into account only temporal adjacency when looking for potential clusters. Time-points were considered to be adjacent if they fell within a 10 ms window (remember that our resolution is 3.9 ms—Section 3.3), as post-synaptic potentials, the kind of potentials captured by EEG recordings, do not last less than 10 ms (Luck, 2014), and therefore the EEG signal can be assumed to be smoothed within that time window.

Given the nested categorical structure of the labels, we paid special attention to data splits among training and testing. We generally took a leave-4-out evaluation approach (see exceptions at the end of this section), which trains on 87.5% of the full original data, leaving out 12.5% for testing. We did not use random folds, which would end up giving unreliable results, because of unbalanced splits at the fine-grained level. Instead we first computed all possible combinations of sample labels to be used as test sets which respect the following criteria: sample labels should be balanced across coarse categories (two people and two places), and the test set should not contain more than one exemplar from each fine-grained categories. An example of a test set could be “*Barack Obama*,” “*Scarlett Johansson*,” “*Eiffel Tower*,” “*South Africa*,” with different labels depending on the analysis: “people,” “people,” “places,” “places” for the coarse-grained analysis, and “politician,” “actress/actor,” “monument,” “country” for the fine-grained one. This procedure can be considered as a balanced implementation of the ShuffleSplit data splitting technique (Pedregosa et al., 2011; Varoquaux et al., 2017).

Given that word length was not strictly controlled across categories in stimuli selection, giving rise to different average word lengths for each category (see Section 3.1 and Supplementary Materials), word length could in principle act as a confound in classification, where categories are used as target labels. In order to control for its effect, we adapted to our classification analyses the confound variable control procedure presented in Chyzhyk et al. (2018). This method was specifically validated by the authors in the case of neuroscience classification analyses where an individual variable has to be controlled, showing that it avoids both overly pessimistic and optimistic accuracies (Chyzhyk et al., 2018; More et al., 2020). The intuition it follows is that, to control for a potential confound in classification, given a large enough pool of candidate train-test splits, it is enough to select and use only the splits where, in the test sets, the outcome is independent from the confound: with this procedure, the classification analysis provides an evaluation of whether the brain data determines successful prediction beyond the confounding effect. In our setup, controlling for word length in this way is straightforward since, as discussed above, we generate as candidate train-test splits all the possible balanced combinations of stimuli. First, for each possible candidate test set, we transform stimuli and target labels (the categories' nouns), that are categorical variables, to numeric values: words become word lengths, and target labels become the average word lengths of the label's entities (as reported in the Supplementary Materials; e.g., “Madonna” is encoded as 7, and “politico” is encoded as 13, the average of all politicians' names in our set of stimuli). Then, we compute the Spearman correlation (indicated by ρ) between the encoded stimuli and target labels: this quantifies the confounding effect of word length on that candidate test set. Having computed the correlations for all the candidate test sets, we sort them in ascending order by their absolute value. Finally, following Varoquaux et al. (2017), where authors recommend using 50 random train-test splits when working with brain data, we retain as final test sets the 50 candidates having the lowest correlation. In the case of the coarse-grained classification, we could use 50 test sets completely uncorrelated from word length (ρ = 0.0), whereas in the case of fine-grained classification the highest correlation is not 0.0, but still close to it (ρ = 0.162) ^2^.

In order to get insights regarding the structure of the representations of proper names, we further exploited the hierarchical structure of the dataset. In a separate set of analyses, we trained on evoked responses for individuals, and we only tested on those for the fine-grained categories. This can be seen as some sort of transfer learning, looking at whether, and when, the two kinds of representations converge. In this case, the dataset was used in its entirety. Furthermore, since we reckoned that there could be some differences in terms of discriminability between people and places, we also ran the analyses separately first on people only, and then on places. In this case we used a leave-2-out classification setup, corresponding again to a 87.5% train–12.5% test split.

### 3.5. Distributional Semantics Models

One of the key goals of our work is exploiting an array of recent, cutting-edge computational models of semantics in order to isolate semantic information in evoked responses in the brain, at the level of unique, individual entities. We selected a set of models which can be connected as transparently as possible to cognitive theories of the semantics of individual entities. At the broadest level, we use three kinds of models. All of them represent individual entities as vectors, but the way in which these vectors are created differs significantly across models.

The first kind of word vector models relies exclusively on distributional information about words as observed in linguistic corpora (BERT Devlin et al., 2019, ELMO Peters et al., 2018, Word2Vec Mikolov et al., 2013; see Section 3.5.1). Within this family, all models follow, with various degrees of sophistication, the distributional principle, according to which words which are found in similar contexts have similar meaning. With respect to the way they represent individual entities, they can be understood as putting them on the same level as all other words—individual entities are not given a special treatment, and their representation follows the same pathway as that of all other words. Distributional models have been often taken as models of human semantic memory, both because of principled reasons (they embody the assumption that word meaning can be retrieved from their use, and that it can be defined in a distributed fashion, across various dimensions) and because of their empirical success at modeling human data.

The second type of models (TransE Bordes et al., 2013; see Section 3.5.2) comes from a very different tradition in artificial intelligence, that of ontologies and structured representations of entities (Guarino, 1995; Ji et al., 2021). Representations of these kinds follow a distinct principle, that of creating models of the world that an artificial intelligence agent could use in an external environment (Guarino, 1995), and have most commonly taken the form of knowledge bases—graphs where entities (both unique and generic) are the nodes, and the links among nodes capture their relationships. In this case, entities—and individual entities in particular—are at the very core of the representational structure.

The third kind of models (LUKE Yamada et al., 2020b, Wikipedia2Vec Yamada et al., 2020a; see Section 3.5.3) is a combination of the two approaches presented above, and it has recently received much attention in NLP because it promises to overcome the limitations inherent to each methodology: for distributional models, the lack of precise entity-level information; and for knowledge bases, their structural rigidity and their difficult integration with generic linguistic knowledge, as well as their costly creation and maintenance (Peters et al., 2019; Sun et al., 2020b; Yamada et al., 2020b). We will call these models, as is commonplace in the NLP literature, entity-aware embeddings. From a cognitive point of view, these computational models may be interpreted as implementing the intuition that individual entities are represented at a separate level than generic entities. Some models introduced this idea in cognitive psychology (Bruce and Young, 1986; Burton and Bruce, 1992; Young, 1999), stating that individual entities bearing a proper name have a dedicated identity node, which is then integrated, at a later stage, with generic semantic knowledge.

For all models we use the version trained on English. This is the language over which the models were developed originally, because of resource availability—as a matter of fact, importantly, for most models the Italian version is not available at all. Also, in terms of performance on NLP tasks in different languages, English consistently presents the best performances available (Bender, 2011; Pires et al., 2019). We used English vectors for many reasons. First of all, our stimuli can be considered to be largely language-independent (Van Langendonck and Van de Velde, 2016), at least across the two languages involved in our experiment (Italian, the language in which the experiment was carried out, and English), and strongly referential in nature: the referents of proper names are specific people and places in the world, which only require to be familiar with them (Kripke, 1972), and the category nouns that we employed (occupations and type of place) are shared across Italian and English cultures. The level of representation of interest is exclusively semantic, thus ruling out orthographic and phonetic language-specific phenomena.

To support our argument, we checked empirically whether using Italian models, instead of English models, would give rise to relevant differences in decoding performance. We ran the analyses with the models available also in Italian (Word2Vec, Wikipedia2Vec and BERT base), and then compared results across languages with a two-tailed non-parametric Wilcoxon statistical significance test, correcting for multiple comparisons using Benjamini and Hochberg (1995)'s False Detection Rate (FDR) procedure, which is a standard procedure in both computational linguistics and neuroscience (Groppe et al., 2011; Dror et al., 2017). Difference in scores was not significant for BERT (*p* = 0.97), whereas it was significant for Word2Vec (*p* = 0.043, with the Italian model performing better—average Italian model accuracy: 0.619, average English model accuracy 0.595) and Wikipedia2Vec (*p* = 0.017, where the opposite was true—average Italian model accuracy: 0.609, average English model accuracy: 0.639; cfr. **Figure 8**). Given that results did not show a consistent pattern of advantage for one language or the other, and that most of the available models were trained on English data, we report and discuss results for the models trained on English.

#### 3.5.1. Static and Contextualized Distributional Models

Distributional models can further be subdivided into static and contextualized. Static models, such as Word2Vec (Mikolov et al., 2013), work at the level of individual lexical items. They are essentially vector spaces, where each vector captures the meaning of an individual word. Contextualized models, such as the widely used ELMO (Peters et al., 2018) and BERT (Devlin et al., 2019), are more recent. They instead focus on sentences, and not on individual lexical items (Camacho-Collados and Pilehvar, 2018). Given a sentence, their goal is that of creating representations of the words contained in the sentence which reflect their current idiosyncratic, context-dependent meaning. Contextual distributional models have been generally shown to improve on static models on most tasks in Natural Language Processing (Rogers et al., 2020), but it should be noticed that they are dramatically more complex than static models (Lenci et al., 2021).

As our static distributional model, we choose Word2Vec, a very well-known model, which has also been shown to be a good model of human semantic memory (Mandera et al., 2017). Word2Vec vectors are created by training a feedforward neural network on a word prediction task. In the case of the model we use here, it is called the “skip-gram” task (Mikolov et al., 2013). It requires to learn to predict whether, given a word in a sentence and another target word from the vocabulary, the target actually comes from the actual set of words surrounding the query, or whether it was randomly sampled among the words not appearing in the window. In this experiment we use a model pre-trained by the authors of the original papers on a corpus of news articles, where individual entities have been marked as individual words so that they end up having their own vector. The vectors were created by the authors of Mikolov et al. (2013), optimizing the learning parameters and the dimensionality (1,000) for the representation of entities.

There is a very large number of contextualized models, often specialized for specific NLP tasks. We chose to use two of the “basic,” vanilla models (which are in any case quite complex) on top of which most of subsequent research has been built, ELMO (Peters et al., 2018) and BERT (Devlin et al., 2019). Despite being created for generic language processing—actually, the adaptability of the word vectors they create is one of the reasons of their success—these models can in most cases compete with specialized models in terms of performance, and they are very often used as a strong benchmark. ELMO is a bidirectional LSTM neural network, which learns to predict a given word conditioned on the previous words, as well as the next ones. BERT, instead, adopts the Transformer architecture (Vaswani et al., 2017), which relies heavily on the computational mechanism of attention (Lindsay, 2020), in order to encode a sentence into a set of vectors capturing both lexical and contextual meaning and structure. Importantly, BERT is a deep architecture (it exists in two flavors, one with 12 layers and one with 24 layers), with different layers encoding—at least partially—different kinds of linguistic information (Rogers et al., 2020).

One of the challenges posed by contextual models, as opposed to static word vectors, is that they leave many free choices to the experimenter when it comes to extracting the vectors: which layer to use; whether to use vectors for word mentions, or instead those for full sentences; what model dimensionality to choose. For both ELMO and BERT, we follow a methodology proposed in Bommasani et al. (2020) and refined in Lenci et al. (2021), which has been show to capture effectively lexical meaning. We extract many vectors for separate mentions of each proper name, and then average them. The mentions are taken from the Wikipedia pages of each individual entity, in order to encode definitional information about the entities themselves. From each Wikipedia page we take up to the first 32 sentences. We experimented with various layers, but finally we decided to use an average of the final layers, as in Lenci et al. (2021) (the last four for BERT, and the last one for ELMO), since they have already been shown to perform well with neural data (Jat et al., 2019). For BERT we used Huggingface's Transformers implementation (Wolf et al., 2020) and the original pre-trained weights, both in their so-called “base” and “large” versions (1,024 and 2,048 dimensions, respectively). For ELMO we used the pretrained original weights (1,024 dimensions) and the AllenNLP implementation (Gardner et al., 2018).

Our method for extracting static representations from contextualized models has not been tested specifically on entities, but only on common nouns. Therefore, we validate it by measuring its performance on WikiSRS, a similarity and relatedness task (cfr. Section 2.2) created specifically for entities. This benchmark was introduced in Newman-Griffis et al. (2018), and it was recently used for the evaluation of contextualized models (Chen et al., 2019). The dataset was created by crowd-sourcing similarity and relatedness judgments for 688 pairs of named entities. To evaluate the performance of our vector extraction methodology, we followed the procedure of Chen et al. (2019): first we obtained the vectors for the entities appearing in WikiSRS; then we computed the pairwise cosine similarities between vectors corresponding to the dataset's pairs; and finally we looked at the Spearman correlation between model similarities and human judgments. We carried out separate evaluations for the two portions of the dataset—once for similarity and another for relatedness. As a baseline, we computed the scores obtained when using the vector extraction methodology proposed in Chen et al. (2019), which differs from ours in two respects: first, they only employed the first sentence from Wikipedia as input for the creation of the entity vector; and second, for BERT, as entity representation, they used the special [CLS] token, used by BERT to represent the whole input sentence. For BERT large, our methodology improves on the baseline for relatedness from ρ = 0.297 to ρ = 0.423, and for similarity from ρ = 0.424 to ρ = 0.491. For BERT base, the model's fit with relatedness scores improves from ρ = 0.287 to ρ = 0.375, and for similarity from ρ = 0.401 to ρ = 0.468. For ELMO, the baseline score for relatedness is ρ = 0.372, while with our method it improves to ρ = 0.399; and for similarity, correlation goes from ρ = 0.424 to ρ = 0.4399. We take this consistent pattern of improvements to confirm the validity of our proposed methodology for the extraction of static entity vectors from contextualized language models.

#### 3.5.2. Knowledge-Base Models

As a knowledge base model, we used vectors obtained using TransE (Bordes et al., 2013), a method to translate a knowledge graph into a set of entity and relationship vectors (we only used the entity vectors). Roughly, TransE starts from random vectors, and goes through the knowledge base, optimizing along the way entity and relation vectors. Vectors are tuned so that, given a triplet of entity_1_, entity_2_ and a relation holding between the two, the sum of the vectors for entity_1_ and the relation should return a vector which is among the nearest neighbors of entity_2_; and the opposite for randomly sampled negative relationships. We employed a pre-trained model, published in Han et al. (2018), which was trained on WikiData, an open knowledge base that can be seen as a structured sibling to Wikipedia (Vrandečić and Krötzsch, 2014). The final vectors have 100 dimensions.

#### 3.5.3. Entity-Aware Models

Finally, as entity-aware embeddings, we use both a static and a contextualized model, built on top of Word2Vec and BERT, that were shown to both improve on their basic version with respect to entity-related tasks in NLP. The first one is called Wikipedia2Vec (Yamada et al., 2020a), and the second one is LUKE (Yamada et al., 2020b). We chose these two models because they both adopt a conceptually similar approach, and they use the same training data, which reduces possible confounds—the English text of Wikipedia, complemented by the underlying graph of the hyperlinks contained in each page. Both models modify their basic training regimes, storing and processing separate representations for individual entities and common nouns. Their strategy consists of exploiting hyperlinks in Wikipedia pages as annotations of entity mentions, using these mentions for an additional entity-specific task which is added to the training. In this task, given an entity mention in a sentence within a Wikipedia page, the model has to learn to predict other entities found in that sentence. We use the pre-trained versions of the models published by the authors. Wikipedia2Vec comes with 500 dimensions, and was trained from scratch with a window size of 10 words and 15 negative examples, for 10 iterations. The entity-specific task is a skip-gram prediction task for mentions of entities. LUKE, both in the base and large versions, has the same dimensionality as BERT (1,024 and 2,048) and was trained on top of pre-trained BERT weights, which were used as initialization weights for the part of the model dedicated to generic word representations. Representations for the individual entities are learnt with the same masked language modeling objective of BERT, just applied separately to entities. For LUKE, which works in practice as a contextualized language model, we follow the same word vector extraction procedure validated in Section 3.5.1.

### 3.6. Decoding to Distributional Word Vectors

In order to find out whether we can distinguish between evoked responses at the finest level of granularity, that of individual identities, we exploit our entity vectors as mapping targets. In other words, for each entity, we use a regularized linear regression model to learn to predict the true values of the dimensions of each entity vector from the corresponding EEG representation of that entity.

As inputs (i.e., as our EEG representations) we use whole epochs, from 100 to 1,200 ms after stimulus onset, which we collapse to one vector, so that for each evoked response to a name or a noun we have one vector. To learn the mapping, we use Ridge regression with default parameters, following recent work (Pereira et al., 2018; Sassenhagen and Fiebach, 2020), implemented in the Python package Scikit-learn (Pedregosa et al., 2011).

We adopt for evaluation the pairwise evaluation approach proposed by Mitchell et al. (2008). This is a leave-2-out training regime, repeated for all possible pairs of stimuli. Note that the model has to learn to predict the true value of each vector dimension for entities which are completely unseen during training—a form of zero-shot machine learning task, which requires the model to isolate and exploit at test time the signatures in the brain of semantic processing of individual entities.

In pairwise evaluation, at test time the model first predicts two entity vectors, $\hat{\vec{e_{1}}}$ and $\hat{\vec{e_{2}}}$, using the corresponding evoked responses; then the respective Spearman correlations to the original entity vectors $\vec{e_{1}}$ and $\vec{e_{2}}$ are computed. At the end there will be four correlation measures, two of which are for the matching vectors and are expected to be, taken together, higher than the correlations for the mismatched vectors. The decoding accuracy evaluation is based on this expectation, in that it is considered successful, with *accuracy* = 1, if $\rho \left(\vec{e_{1}},\hat{\vec{e_{1}}}\right)+\rho \left(\vec{e_{2}},\hat{\vec{e_{2}}}\right)>\rho \left(\vec{e_{1}},\hat{\vec{e_{2}}}\right)+\rho \left(\vec{e_{2}},\hat{\vec{e_{1}}}\right)$; else, decoding is considered unsuccessful, and *accuracy* = 0.

We controlled for statistical significance using a one-sample, one-tailed Wilcoxon test, because of the minimal assumptions made regarding the distribution underlying the scores (Grootswagers et al., 2017). We also applied FDR to control for multiple comparisons, given that we were running statistical analyses for eight models.

With respect to word length, we could not apply straightforwardly the confound control procedure presented in Section 3.4, since decoding to word vector is not a classification task. However, we point out that, in this case, word length should not even be considered a confound variable for principled reasons: word vectors do not encode any information about word length at all. To show that this is the case, we compute the Spearman correlations between pairwise vector similarities and the matched pairwise differences in character length among words (the intuition being that, if word vectors encoded information regarding word length, their distance in vector space should correlate with the number of additional letters required to match two words in length). Results confirm that word length should not be considered as a confound variable for the word vector decoding analyses: for all models except one we found extremely low correlations (in all cases, ρ <0.08); the only exception is LUKE, where correlation is anyways quite mild (LUKE base: ρ = 0.144, LUKE large: ρ = 0.326).

**Figure 2:**
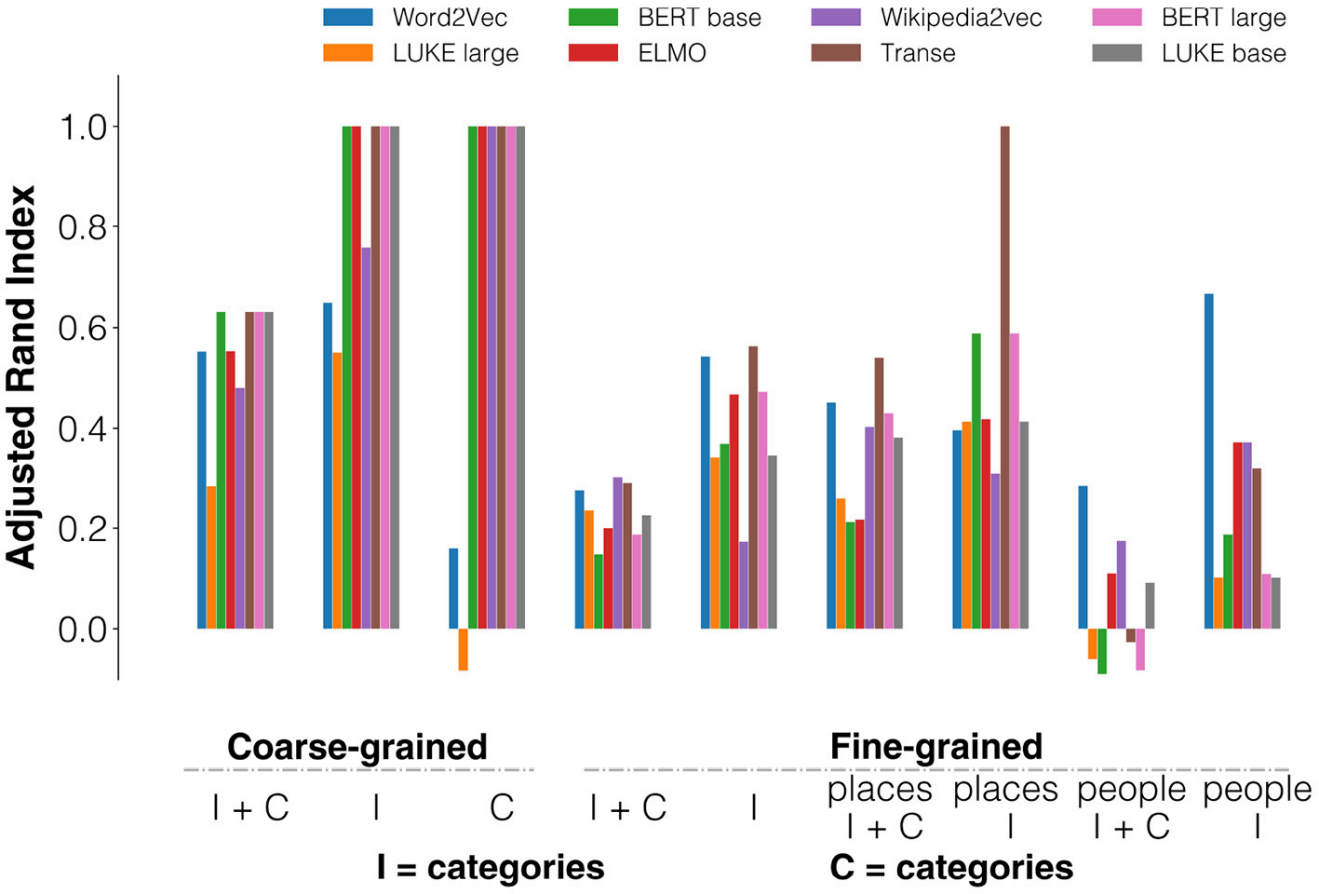
Clustering results on word vectors, with various possible assignments of categorical labels. We test the performance of each model on a completely unsupervised clustering task. In this evaluation, we measure to what extent word vectors can be clustered according to their category, evaluating separately coarse-grained and fine-grained categories. The evaluation metric that we used, Adjusted Rand Index, involves a correction for chance, so results above 0.0 show some sensitivity to categorical structure. We report results for all possible combinations of stimuli: clustering using only the vectors for individuals; only the vectors for categories; both categories and individual entities; people and places separately. Most models perform above chance in all clustering tasks.

In order to prove that the word vectors actually captured the kind of semantic information regarding entities that we wanted to isolate in EEG data, we carried out some clustering analyses as a validation procedure, as is customary with distributional models (Almuhareb and Poesio, 2004; Baroni and Lenci, 2010; Lenci et al., 2021), reported in Figure 2. We measure to what extent we can cluster word vectors according to their coarse-grained and fine-grained semantic class, when considering individuals only, categories only, or both individuals and categories; and when restricting the analyses to people and places only, or using both people and places together. As a clustering algorithm, we use K-Means, a common choice in computational linguistics (Lenci et al., 2021). As an evaluation measure we use the adjusted Rand Index, which looks at all possible pairs of samples, measuring how many pairs are correctly assigned to the same or a different cluster, then correcting for chance (Hubert and Arabie, 1985). Both for the clustering algorithm and the evaluation measures we use their Scikit-learn implementation (Pedregosa et al., 2011).

The results show that clustering is above chance (>0) for almost all models. Discrimination is easiest for coarse categories, and particularly so when individuals and categories are clustered separately. Performance is above chance for most models in all possible labellings, for fine-grained categorical labels as well. The toughest discrimination is the one where both individual and category vectors for people are used, and in this respect others have already found that social categories for people are not well captured by distributional models (Westera et al., 2021). Also, in general, performance worsens when clustering vectors for individuals and categories together, as already observed by Gupta et al. (2018). Overall, however, indicate that the distributional models encode the categorical structure that we need in order to use them for decoding.

## 4. Results

### 4.1. Coarse-Grained Semantic Category Classification

**Figure 3:**
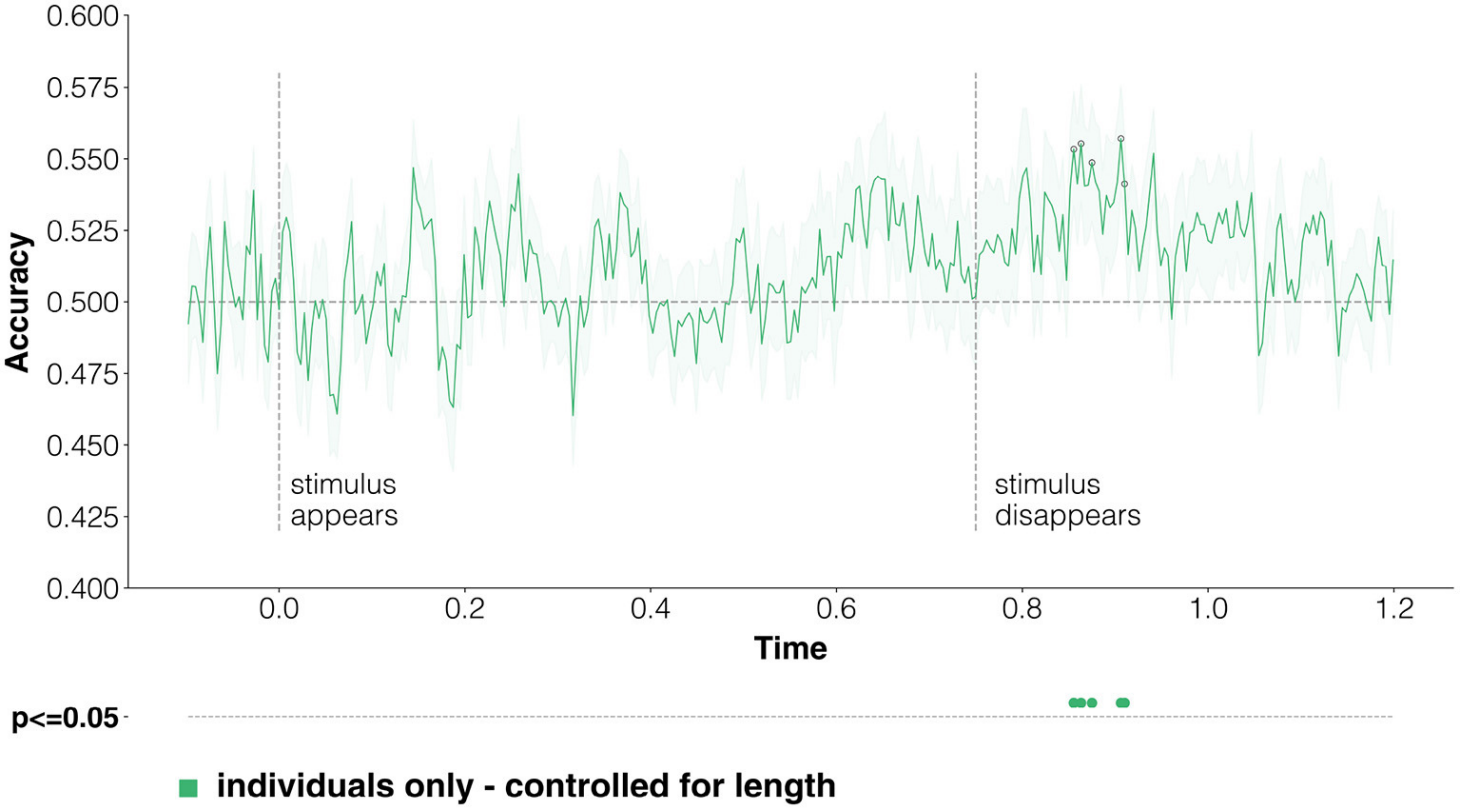
Classification of coarse-grained categories from individuals. We run a time-resolved binary classification on the EEG data. For each time point, we learn to classify evoked responses according to their coarse semantic category (either person or place). We control for word length employing the test sets where the correlation between labels and stimulus length is lowest (see Section 3.4). The green line is the average of results across the 33 participants, and the shaded areas correspond to the standard error of the mean. The random baseline is at 0.5, given that this is a binary classification problem, and is represented by a dotted horizontal line; we also plot as dotted vertical lines the time-points when stimuli appear and disappear. Statistically significant points (*p* < 0.05 corrected for multiple comparisons by TFCE; see Section 3.4) are reported both on the averaged lines and, to make them easier to read, below the *x* axis.

We report in Figure 3 the averaged results for the time-resolved classification of coarse-grained categories, where we classify evoked responses at time *t* into two classes, either person or place. Classification accuracy raises intermittently above the baseline level from around 150 ms after stimulus onset, which is compatible with visual word recognition processes starting at around 150 ms (Carreiras et al., 2014; Ling et al., 2019)—but never reaches significance while the stimulus in on screen. We interpret this as an effect of the variable control procedure for word length described in Section 3.4, given that it is in this time range that semantics and word-reading processes can get confounded. Scores reach statistical significance later on, starting at around 800 ms, indicating that it is possible to decode coarse-grained categories of individual entities from EEG data during the mental imagery task.

**Figure 4:**
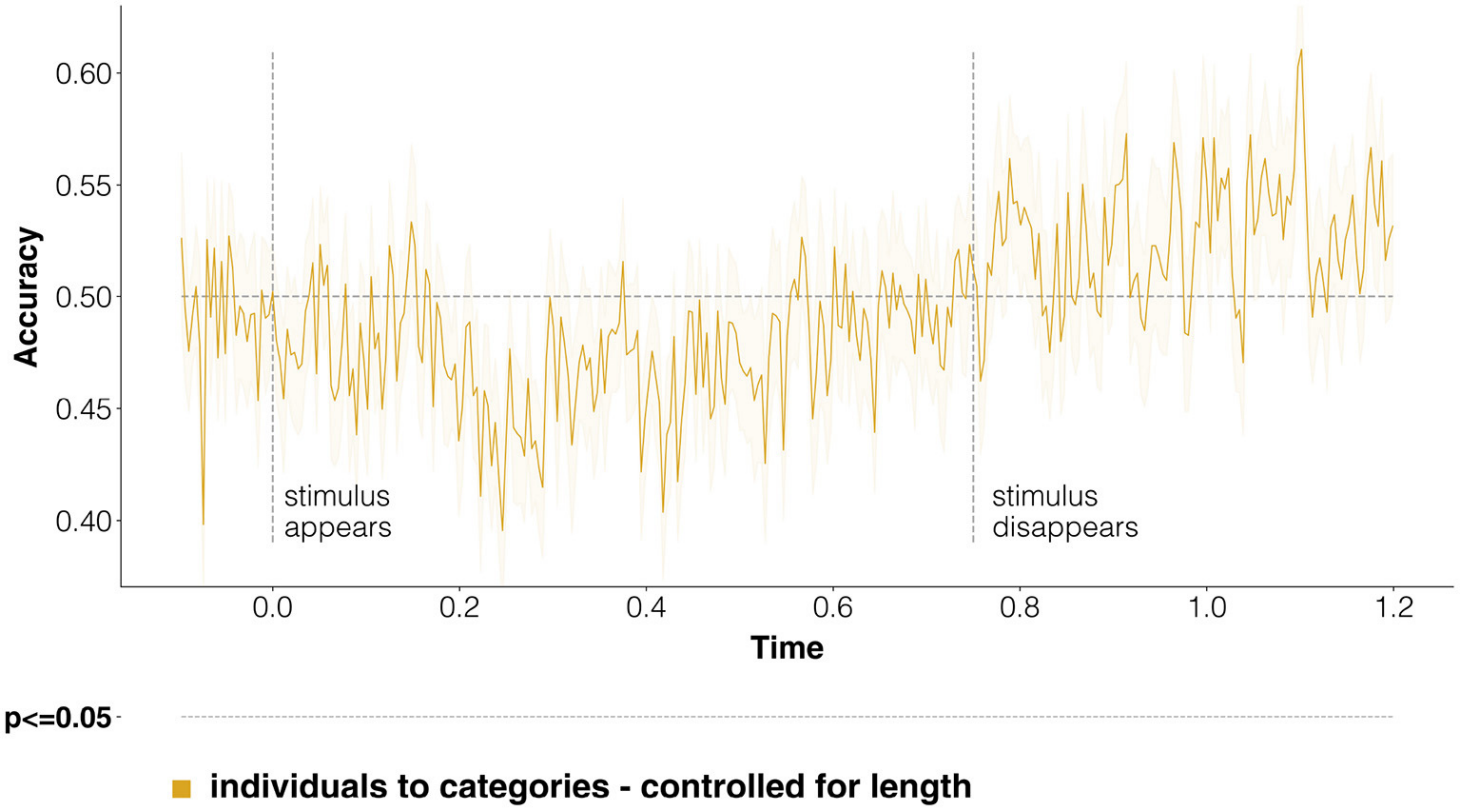
Classification of coarse-grained categories, transferring information from individual entities to categories. Here we report time-resolved classification scores, following the same structure as Figure 3. However, in this case we look explicitly at whether discriminative representational information is shared across individuals and entities, training on individuals, and testing on categories. The results show that this is not the case: scores go past the random baseline only after the stimulus disappears, and never reach statistical significance.

One of our objectives was trying to understand whether, and when, semantic information about the coarse-grained class is shared in brain processing between proper names and the names of the categories these individual entities belong to. In Figure 4 we report classification scores obtained when training on individuals, but then testing on categories only—effectively looking at how much information about coarse-grained semantic categories can be transferred from instances of categories (the individual entities) to the categories themselves. Although statistical significance is never reached, decoding performance goes above baseline after 800 ms, during the mental imagery task. The lack of significant decoding accuracies in Figure 4, which are instead reached when using only evoked responses to stimuli at the same ontological level (instances, instead of categories; Figure 3), suggests that coarse-level semantic information does not seem to be strongly shared between entities and categories.

### 4.2. Fine-Grained Semantic Category Classification

#### 4.2.1. Aggregate Results

**Figure 5:**
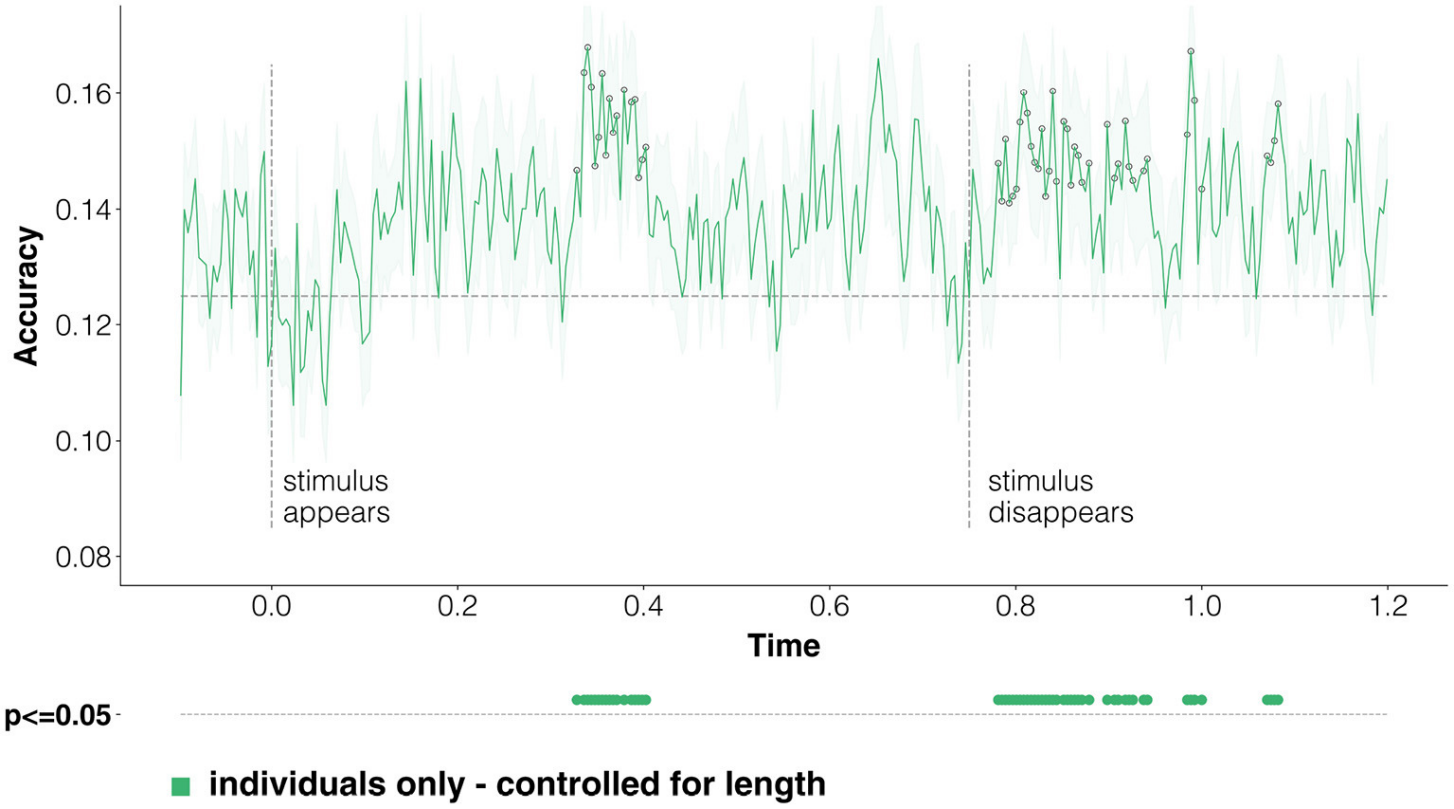
Classification of fine-grained categories from individuals. We run a time-resolved multi-class classification analysis, trying to decode at each time point the fine-grained semantic category of the stimulus. Figure structure is the same as in Figure 3, but here the random baseline is at 0.125, since there are eight possible categories (four for people and four for places). Scores are statistically significant between 300 and 400 ms, and from 800 to 1,100 ms.

When it comes to classification of fine-grained categories, as it can be seen in Figure 5, classification accuracy is again above chance starting after around 150 ms. It reaches statistically significant discriminability between 300 and 400 ms, then it drops until 800 ms, when scores start being statistically significant again during the mental imagery task, until 1,100 ms. A peak of classification accuracy close to 400 ms is to be expected, as this has been consistently found to be the time frame where word-level semantic processing happens (Hauk et al., 2006; Simanova et al., 2010; Frank et al., 2015; Sassenhagen and Fiebach, 2020). For instance, the N400, a negative deflection in recorded brain potentials at around 400 ms after stimulus appearance, is considered to be a signature of semantic processing in EEG responses, although it is still debated precisely what semantic process it should reflect (Lau et al., 2008; Kutas and Federmeier, 2011; Rabovsky et al., 2018). Concerning mental imagery, the scores strongly indicate that fine-grained categorical information can be discriminated during this experimental task, converging with the results of Figure 3.

**Figure 6:**
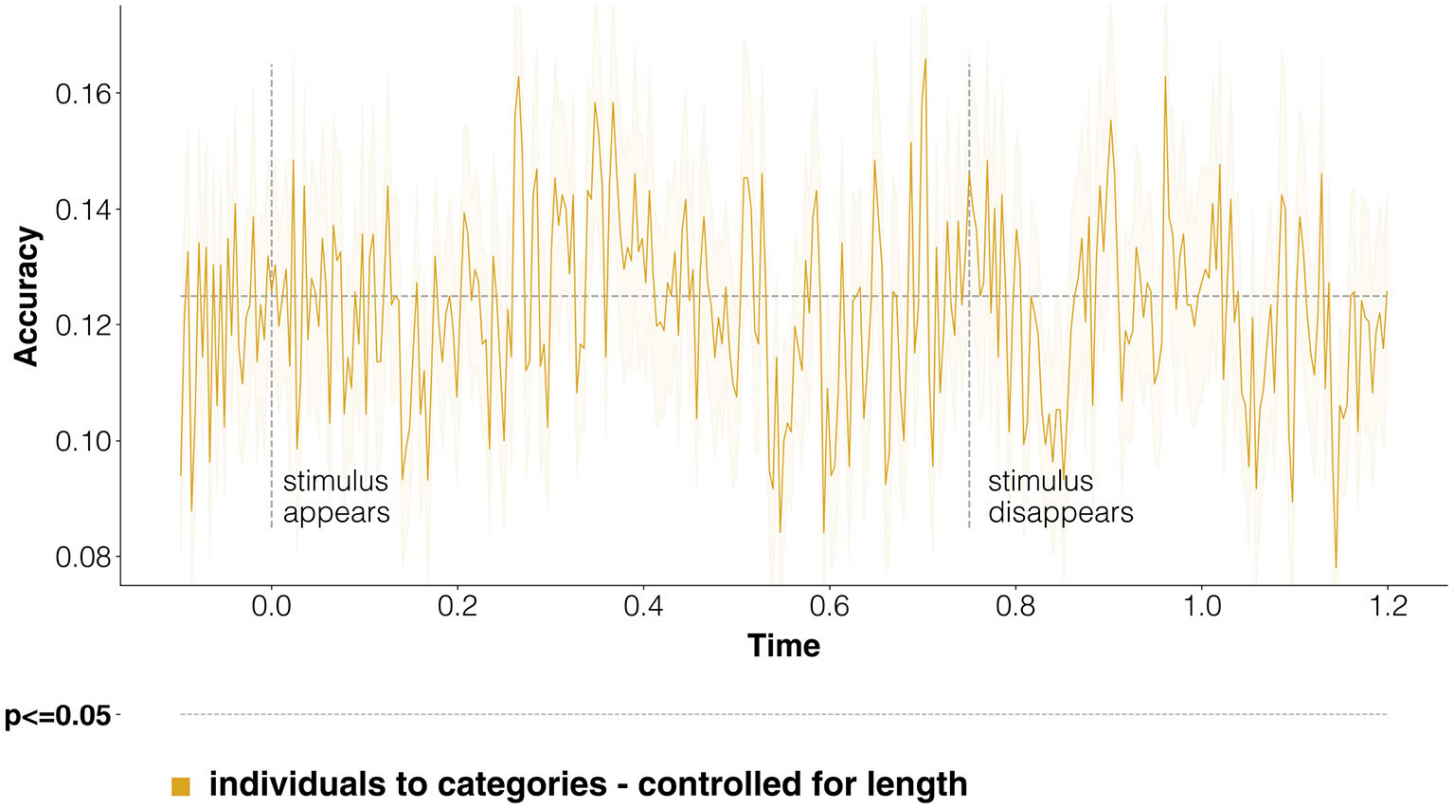
Classification of fine-grained categories, transferring information from individual entities to categories. This figure is the equivalent of Figure 4, just referring to the multi-class classification task involving fine-grained categories as labels. The random baseline is set at 0.125, because of the presence of eight possible classes. Notice that decoding scores follow a very different course with respect to coarse-grained categories. Statistical significance is never reached, confirming that little discriminative semantic information is shared between responses to individuals and categories.

When considering the commonalities between evoked responses for individual entities and categories (Figure 6), we see that decoding accuracy when training on individuals and testing on categories barely makes it past the random baseline at discontinuous time-points, never reaching statistical significance. These results concur with those of Figure 4, in that both seem to indicate that not much in terms of semantic representation is shared across individuals and categories.

#### 4.2.2. Per-Category Results

We also compared, separately, the performance on either people or places. These two coarse-grained categories may, in principle, produce very different results, as their respective fine-grained categories are defined differently, by necessity: for people, based on their occupations; for places, more generically based on their most immediate superordinate category (cfr. Section 3.1).

**Figure 7:**
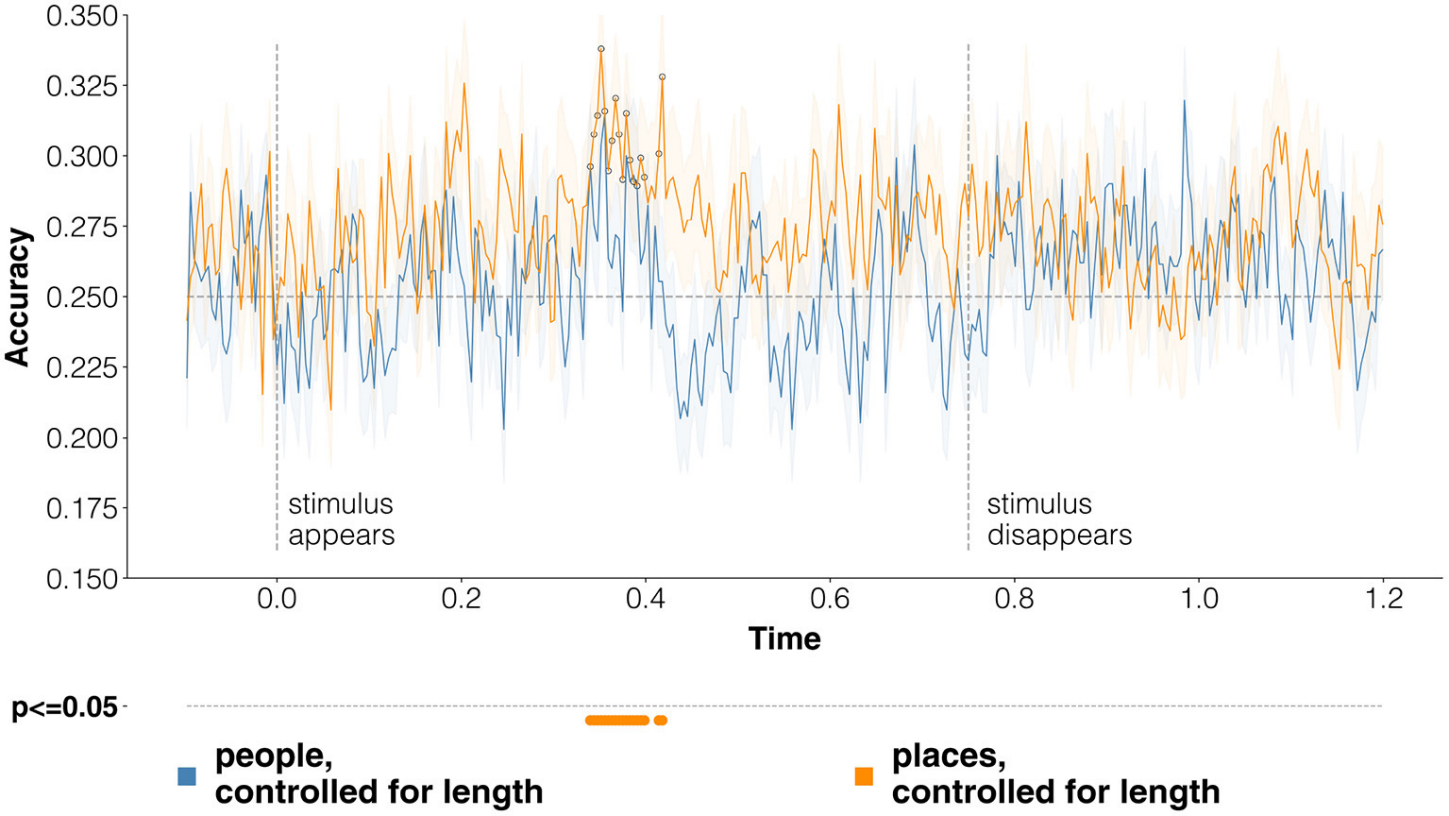
Classification of fine-grained categories, separately for people and places, considering individuals only. We plot classification scores against time, as reported above in Figures 3–6, with the exception that here we restrict our analyses to evoked responses for either people or places. Since there are only four possible classes, random baseline is at 0.25. Interestingly, for people results never reach significance, suggesting that people categories are hard to decode from EEG data. Instead, when decoding fine-grained semantic categories of places, results are strikingly different: classification reaches statistical significance between 300 and 400 ms.

And indeed, the emerging patterns of results go in different directions. Results for both categories are reported in Figure 7. When using only evoked responses to people, classification performance never reaches significance, and is above the chance baseline only shortly at various time-points (around 300–400 ms, 500 ms, 700 ms, and 800–1,100 ms). On the other end, limiting the analyses to fine-grained place categories results in better classification accuracy, reaching statistical significance between 300 and 400 ms (as in Figure 5). These analyses indicate that fine-grained categories are more easily discriminable in our EEG dataset in the case of places—in lay terms, that it is harder to find in the evoked responses separate traces of the semantic distinctions among musicians, writers, politicians and actors than it is when we consider monuments, cities, countries and bodies of water.

It is impossible to reach strong conclusions given the differences in the nature of the fine-grained categories of people and places (see above and Section 3.1), but it can be noticed that these results converge with previous results showing a distinction, within individual entities, between semantic processing for proper names of conspecifics and other kinds of entities such as places (Miceli et al., 2000; Lyons et al., 2002; Caramazza and Mahon, 2003; Mahon and Caramazza, 2009; Fairhall et al., 2014).

### 4.3. Decoding to Distributional Word Vectors

**Figure 8:**
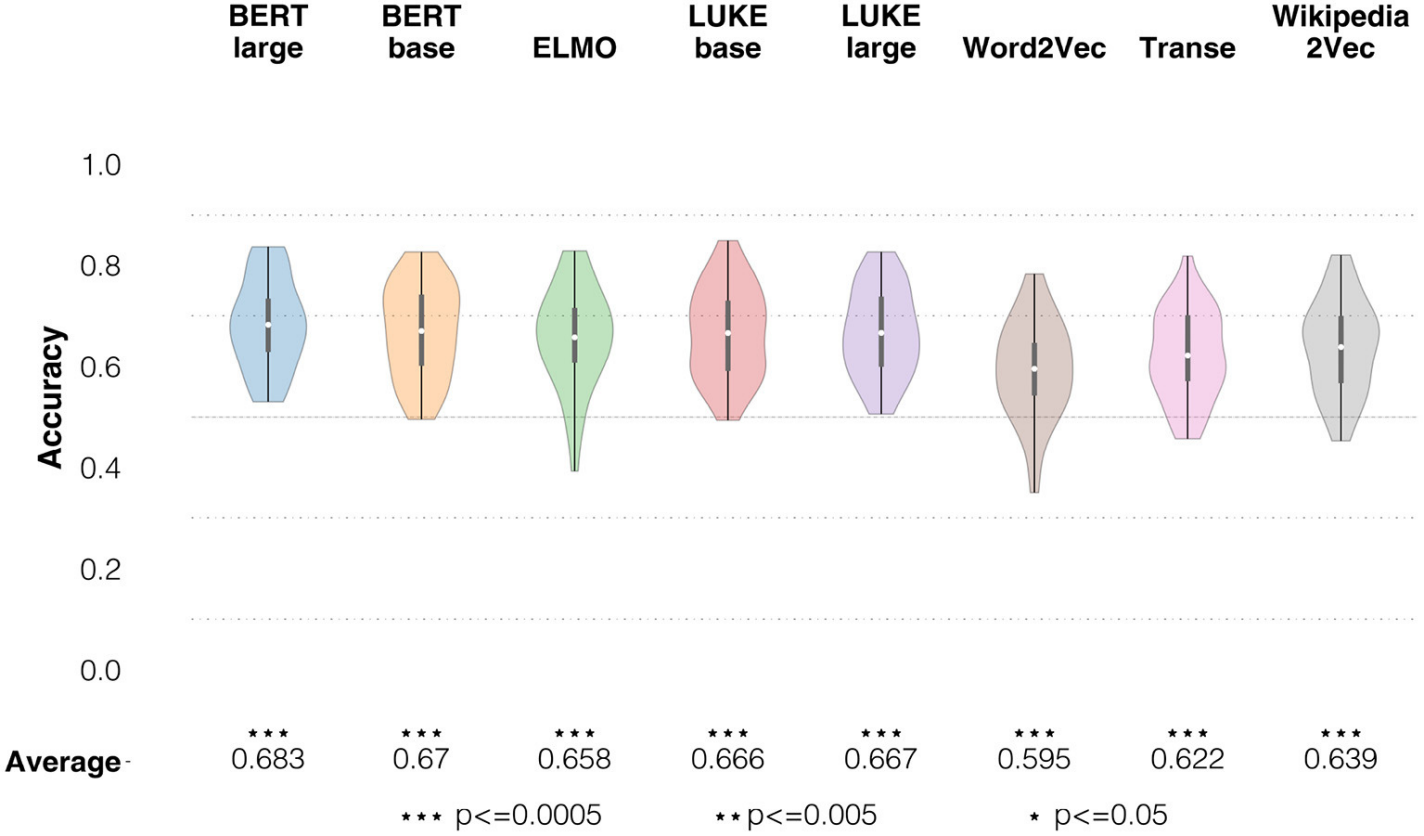
Decoding to word vectors, considering only individual entities. We report the distribution of the scores for 33 subjects when predicting word vector dimensions from EEG data. In this case, we employ evoked responses and word vectors for individual entities. Evaluation is carried out using the leave-two-out methodology proposed in Mitchell et al. (2008). For each computational model, we plot the distributions as violin plots, where the white dots indicate the model average score, and gray bars are used for the 95% confidence interval. We run a one-tailed Wilcoxon statistical significance test against the chance baseline of 0.50. Stars indicate the resulting *p*-value. In general, results are well above random performance, providing evidence that it is possible to distinguish representations of individual entities in the brain, by exploiting computational models of language. Also, contextualized models perform better than their static counterpart.

In Figure 8 we report the results obtained when decoding from evoked responses to word vectors: training and testing is limited to individual entities only, since we are looking at whether it is possible to discriminate among individual entities in the brain.

The best average performance, 0.683, is reached by the largest version of BERT, which in NLP often provides excellent performances (Rogers et al., 2020). Overall, contextualized models show higher accuracies (average scores: *BERT* *large* = 0.683, *BERT* *base* = 0.67, *LUKE* *large* = 0.667, *LUKE* *base* = 0.666, *ELMO* = 0.658), providing better fits than static (average scores: *Wikipedia*2*Vec* = 0.639, *Word*2*Vec* = 0.595) and graph-based models (average score for *TransE* = 0.622). In order to test the statistical significance of results, we compare all possible pairs of models using a two-sided Wilcoxon test with FDR control of multiple comparisons. All contextualized models show statistically significant improvement on Word2Vec (in all cases, *p* < 0.0005); and very similar results emerge regarding TransE, since the difference in scores is statistically significant for all models (BERT base: *p* = 0.0021, BERT large: *p* = 0.0004, LUKE base: *p* = 0.0054, LUKE large: *p* = 0.0085) but ELMO, which is approaching significance (*p* = 0.057). Difference with Wikipedia2Vec is statistically significant for LUKE base (*p* = 0.024), BERT base (*p* = 0.035) and BERT large (*p* = 0.0054).

When comparing contextualized models with one another, statistical significance tests do not provide any *p*-value below 0.05, indicating that no reliable difference among the performances of these models exist; nevertheless, it should be noticed that BERT large is not only the model showing the best average results, but also the only model to get close to significance against other contextualized models (against BERT base: *p* = 0.064; against ELMO: *p* = 0.088; against LUKE large and base: *p* = 0.132), while the p-values for comparisons involving the other contextualized models are well above 0.3. Within static models, statistical significance is only reached when comparing Wikipedia2Vec and Word2Vec (*p* = 0.0062). Taken together, these results also suggest that adding knowledge graph information to word vectors does not make them better matches for their brain counterparts, neither for contextualized nor for static models.

**Figure 9:**
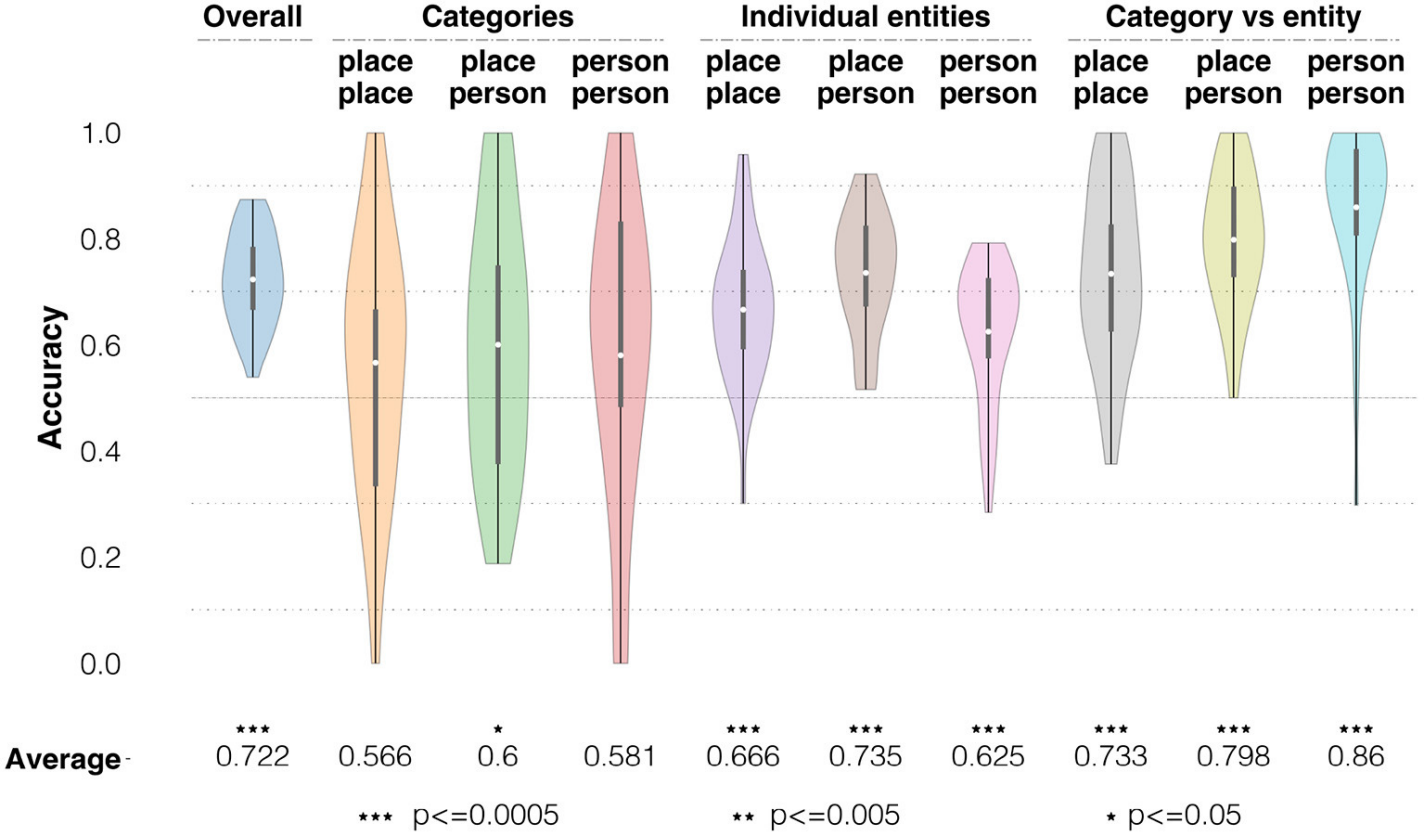
Breakdown of results when decoding to word vectors, considering both individuals and categories. Making the most of the leave-two-out evaluation methodology, which allows to split results according to the semantic and ontological category of the two words, we break down the results for the model with the highest average score, which is BERT large. Each violin plots the distribution of scores for a given combination of categories across subjects. Scores are statistically significant in all cases, except for the case when only categories are used. There, only one set of scores is statistically significant at *p* < 0.05, and the 95% confidence interval always include the baseline, indicating that performance is not reliably above chance. Confirming that individual entities and categories are easiest to discriminate, scores improve dramatically when an individual entity and a category are left out for testing.

In order to better understand how each type of semantic organizational principle (person or place, individual or category) affects decoding scores, and how semantic representations may differ or converge across the various categorizations, we exploit our full set of evoked responses, including both individual entities and categories for training and testing. Doing so while using the leave-two out evaluation of Mitchell et al. (2008), gives us the possibility to break down scores into separate bins, depending on the two evoked responses which compose the test set. We could further subdivide results into accuracies when both test samples are individual entities, or categories, or mixed; and even further, into separate scores for test instances having words referring to two people, or two places, or a mixture of the two. We report in Figure 9 a breakdown of the results, when using BERT large, our best model (results with other models are strongly correlated, on average ρ = 0.93), on the full dataset of individual entities and categories.

As is to be expected, better scores are obtained when comparing, in the pairwise evaluation, one person and one place, with just one exception: representations for people's categories and entities are easiest to discriminate when compared with one another. High performances, well above 0.7, are reached when the test instances are one category and one entity—confirming that representations of entities and categories seem to be segregated in the brain. The worse results come from decodings of categories alone, rather surprisingly given that previous literature has shown that common nouns can be decoded successfully. Importantly, in these cases the 95% confidence intervals always include the random baseline, a sign that, even in the only case where a significant *p*-value is reached (*p* < 0.05, people against places), performance cannot reliably be said to be above chance. In this respect, we are not able to draw conclusions given that our set of categories is small (8 in total). However, we can point out that, when considering that most of the training set is made of individual entities, these results seem to suggest that the decoder could not find much information that could be transferred between the representations of individual entities and their categories.

## 5. Discussion

### 5.1. The Advantages of Bringing Together Two Approaches

Some of the results of our work are primarily of interest from a cognitive neuroscience perspective, whereas others are of interest mainly to practitioners of computational linguistics, NLP and artificial intelligence. Nevertheless, each of these perspectives should be of interest to the other side, and in our experiments we brought them together in order to maximize the reciprocal relevance of, on the one hand, brain data, and on the other, distributional models of word meaning. This interplay of cognitive neuroscience and computational linguistics in our work can be seen in many directions.

First of all, we did not employ specialized experimental tasks, such as semantic priming. These may induce task-related, strategic biases in the results, as argued separately in Wiese and Schweinberger (2011), Wiese (2011), and Adorni et al. (2014), which makes them harder to use for artificial intelligence research. Instead, we aimed to capture snapshots of brain signals for semantic processing of names and their categories that could be aligned with representations of the same names and nouns obtained from models of meaning in artificial intelligence. We achieved this by using a nested set of stimuli and a paradigm which motivates the participants to retrieve and focus mentally on their representations of individual entities and the respective categories.

We then carried out two types of machine learning-based analysis of the relationship between the representation individual entities and that of categories. The first group of analyses, summarized in Figures 3–7, focuses on brain data only, but with results that should have an import on computational models–namely, that categories are represented at a separate level from entities. These constraints should be taken into account when developing computational models of individual entities. In a second set of analyses, reported in Figures 8, 9, we directly aligned brain data and distributional models, using the latter to isolate pieces of semantic information in the brain with great precision, by way of decoding. In this case the connection between the two approaches is particularly evident: computational models find traces of semantic processing of individual entities in the brain, as it can be captured by word distributions in text, without confounds from other linguistic processes such as orthography and morphology; and conversely, measures of the fit of each model with brain data quantify the amount of cognitively-relevant information contained in each distributional semantics model.

### 5.2. Decoding Individual Entities Using Word Vectors

The aspect of this work that is likely to be of the greatest interest to the NLP and AI communities are our results regarding the decoding of responses to individual entities to word vectors.

First of all, we have shown that it is possible to map from brain representations of individual entities to their distributional vectors. So far, this had only been achieved for common nouns (Mitchell et al., 2008; Anderson et al., 2017; Pereira et al., 2018).

These results are all the more surprising because individual entities are semantically much more fine-grained than generic entities, and their meaning is traditionally taken to be determined to a much greater extent by their real world reference, rather than their distributional behavior (Kripke, 1972). Other proposals argue that the meaning of proper names is determined socially (Jeshion, 2009). Neither type of information is easy to extract from text alone, although much research on multimodal models can be seen as providing a framework for the direct reference issue (Bruni et al., 2014), and selected types of text, such as social media posts and novels, may provide enough data for the extraction of social networks from word distributions (Dunbar et al., 2015; Hutchinson and Louwerse, 2018).

Another result of interest to the community of computational linguistics and NLP is the fact that our decoding results, and the associated statistical significance tests, provide some evidence that contextualized models represent individual entities in a way that is closer to what the brain does, compared with statistical distributional models or knowledge-graph methods (see Section 4.3). These results add to a recent body of work which also finds this advantage for contextualized models for both sentence processing decoding and common noun decoding (Jat et al., 2019; Schwartz and Mitchell, 2019; Sun et al., 2020a; Anderson et al., 2021), and worse performance for category-based models (Sassenhagen and Fiebach, 2020). However, we did not intend to provide an in-depth evaluation of distributional models with respect to their ability to capture semantic information about individual entities. Extensive evaluations of distributional models regarding their knowledge of entities are becoming increasingly important in NLP, testing the models' abilities to capture factual and relational knowledge (Petroni et al., 2019), similarity among entities (Newman-Griffis et al., 2018), information about entity types (Choi et al., 2018), or co-reference (Sorodoc et al., 2020) and disambiguation (He et al., 2013) phenomena (for a comprehensive set of tests, see Chen et al., 2019). In this work, however, we only used widely adopted models, whose performance rank among the best in their family (although not necessarily the best), making as clear as possible their theoretical assumptions with respect to cognitive theories of representations of entities. We assume that the core result patterns would translate also to the other models in the family. In this respect, the most similar approach to ours is that of Westera et al. (2021), where the authors show, by using just one model, Word2Vec, that better representations for categories of entities can be obtained by averaging exemplars instead of acquiring separate vectors for the categories, by evaluating the vectors on a set of human judgments.

One surprising result is that the decoding performance for common nouns referring to categories, that we reported in Figure 9 in the second, third and fourth violin plots from the left, is low. Decoding is statistically significant only when the categorical distinction between people and places makes the task easier—but even in that case, the 95% confidence interval includes the baseline, indicating that performance is not reliably above chance.

Part of the reason is clearly that much lower accuracy is obtained for decoding from EEG than for decoding from fMRI. Also, we do not have many categorical stimuli. This leaves unanswered whether our conclusions would apply also to other kinds of categories often employed in computational linguistics. To our knowledge, only one other kind of individual entities has been studied in cognitive neuroscience—brand names, whose closest notion in computational linguistics is that of organizations. Although very few studies exist (Gontijo et al., 2002; Crutch and Warrington, 2004; Cheung et al., 2010), proper names of brands seem to have similar brain processing to those of famous people; if this is the case, then we would expect that, in principle, the two should behave similarly in an experimental setup like ours.

A different question is why should categories of people be harder to decode than categories of places in classification analyses (Figure 7). We do not have a full story here, but we will just point out that social concepts are an idiosyncratic type of category, falling in between abstract and concrete concepts (Anderson et al., 2014; Rice et al., 2018; Conca et al., 2021), and that we found, consistently with the literature (Westera et al., 2021), a similar pattern of results in our clustering analysis for distributional models (Figure 2).

### 5.3. Methodological Innovations

From a methodological point of view, it is important to notice that we always employed a zero-shot paradigm: i.e., we always used as test samples the evoked response to stimuli not seen at training time, a procedure which ensures that we were not overfitting (Varoquaux et al., 2017; McCartney et al., 2019). As argued in Mitchell et al. (2008) and Pereira et al. (2018), this approach results in robust model which is expected to generalize well to other unseen stimuli with similar semantic properties–common nouns in their case, proper names of invididual entities in ours.

A second important characteristic of our approach is that we did not employ images as stimuli–the most common choice in EEG research for decoding studies– but words. We did this knowing that using written words results in lower classification scores compared to either spoken words or images (Simanova et al., 2010). The advantage of written words is that we could compare individual entities belonging to different coarse-grained categories (people and places), avoiding confounds such as face recognition processes (Rossion, 2014) or low-level image features (Rossion and Caharel, 2011). The fact that we nevertheless achieved above chance discrimination can be explained by the fact that we made sure to engage semantic processing by adding two experimental tasks: the mental imagery task, and the categorical question.

Regarding the mental imagery task, results in Figures 3, 5, 8, 9 suggest that it can be used, when induced by written stimuli, as a reliable way to capture semantic processing in the brain. In previous work (Shatek et al., 2019), decoding from mental imagery proved difficult to accomplish. An explanation for this variability in results, also discussed in Shatek et al. (2019), could be that whereas stimulus (word or image) processing is tightly time- and phase-locked to the stimulus presentation (it is strictly speaking “evoked” Bastiaansen et al., 2011), the mental imagery task is less bound to the stimulus presentation: the subjects do not have a clear cue or stimulus to constrain them, and the recorded signals are therefore potentially prone to showing more variance across subjects, which could ultimately make aggregated accuracies lower (Dijkstra et al., 2018; Shatek et al., 2019). Our results suggest that it is possible to induce mental imagery from written words and successfully decode it; however, how to most effectively time-lock mental imagery to experimental stimuli, as well as its temporal dynamics and the effect of imageability (Rofes et al., 2018) remain open questions.

An important feature of our experiment is that, by using written stimuli, we could directly compare semantic processing for individual entities and categories. Nevertheless, our results with transfer classification analyses (Figures 4, 6) should be interpreted with caution, because of two reasons: first, the fact of having used written words as stimuli may still induce differences in representations; second, the two levels of semantic specificity and social relevance may inherently involve different brain processing (Ross and Olson, 2012).

Regarding the former, we believe that we were able to mitigate the confounding effects of written stimuli through our control procedure for word length in the classification analyses (see Sections 3.4, 4.1), and by using machine learning algorithms which can isolate semantic processing beginning immediately after visual word recognition (Hauk et al., 2006; Penolazzi et al., 2007) and running in parallel to visual word processing. With respect to the latter, we notice that such an issue is inevitable in a setup like ours where we wanted to directly compare two inherently different levels of representation (Dehaene, 1995; Proverbio et al., 2009)—but also that, in cognitive neuroscience, this has been shown to be an approach which can reveal common representational properties across disparate kinds of brain responses (e.g., mapping between visual and auditory modalities King and Dehaene, 2014; Leonardelli et al., 2019). A different experimental paradigm could have avoided this concern; however, our analysis, due to its straightforwardness, at least suggests quite clearly that strong commonalities are not present.

### 5.4. Structural Properties of the Representations of Individual Entities in the Brain

From a neuroscientific point of view, throughout our analyses, we have shown that the evoked responses to individual entities and categories were consistently both hard to bring together when it came to find similarities (Figures 4, 6), and easy to tease apart when the goal was that of distinguishing them in pairs (Figure 9). These results suggest that the representations of individual entities and those of the categories involve limited common semantic processing and information—a position advocated in the past by, among others, Young et al. (1994), Barry et al. (1998), Carson and Mike Burton (2001), Turk et al. (2005), and Germain-Mondon et al. (2011). It remains an open question whether using a different experimental paradigm, and less noisy brain data acquisition methods such as fMRI, may shed some more light over the extent, as well as the location, of the interactions between these two interrelated pieces of semantic knowledge.

## 6. Conclusion

In this paper we explored the representation of individual entities—entities referred to by proper names—both from the point of view of cognitive neuroscience (acquiring data about their representation in the brain and investigating the structure of these representations) and from the point of view of computational linguistics and NLP (investigating the extent to which distributional representations of individual entities can be aligned to their brain representations).

More precisely, we tackled four research questions. First of all, we were able to isolate, for each individual entity, distinctive signatures in the brain (Section 4.3), and to classify them according to both their coarse and fine-grained categories (Sections 4.1, 4.2). We also found that it is difficult to transfer representational information learnt from the evoked responses to individual entities (e.g., *Johnny Depp*) to (the nouns for) the categories to which they belong (e.g., actor; Sections 4.2, 4.3). Finally, we provided evidence that distributional models can be mapped with statistically significant performance onto brain representation of individual entities (Section 4.3). Crucially, we were able to obtain these results using EEG, which has inherently lower signal-to-noise ratio than fMRI, but is cheaper and much more portable. This suggests that EEG can act as a useful source of data to investigate jointly brain and artificial intelligence models of language, even for extremely fine-grained semantic processes.

## Data Availability Statement

The original contributions presented in the study are included in the article/Supplementary Materials, further inquiries can be directed to the corresponding author/s.

## Ethics Statement

The studies involving human participants were reviewed and approved by SISSA Ethics Committee (SISSA-Via Bonomea, 265, 34136 Trieste TS, Italia) the participants provided their written informed consent to participate in this study.

## Author Contributions

AB: conceptualization, methodology, software, formal analysis, investigation, data curation, visualization, and writing–original draft. MP: conceptualization, methodology, writing–review and editing, supervision, project administration, and funding acquisition. Both authors contributed to the article and approved the submitted version.

## Funding

AB is supported by a doctoral studentship from the School of Electronic Engineering and Computer Science, Queen Mary University of London.

## Conflict of Interest

The authors declare that the research was conducted in the absence of any commercial or financial relationships that could be construed as a potential conflict of interest.

## Publisher's Note

All claims expressed in this article are solely those of the authors and do not necessarily represent those of their affiliated organizations, or those of the publisher, the editors and the reviewers. Any product that may be evaluated in this article, or claim that may be made by its manufacturer, is not guaranteed or endorsed by the publisher.

## Footnotes

^1^ https://www.biosemi.com/activetwo_full_specs.htm

^2^Our implementation differs from the original implementation in two respects: first, we use Spearman correlation, a linear measure, whereas in Chyzhyk et al. (2018) the authors ranked and selected candidate test sets based on mutual information, which is non-linear; and secondly, here we have individual trials as candidate test items, while in Chyzhyk et al. (2018) the candidates for testing were whole subjects to be left out.
