# Supplementary material for: Exploring the Representations of Individual Entities in the Brain Combining EEG and Distributional Semantics
Source: Front Artif Intell. 2022 Feb 23;5:796793. doi: 10.3389/frai.2022.796793 (PMC8905499; doi:10.3389/frai.2022.796793)
Supplement: Supplementary file 1 [file Data_Sheet_1.PDF]

Supplementary Material

1 EXPERIMENTAL STIMULI

We report here two tables detailing the experimental stimuli, one for each coarse-grained category of individual entities (people and places). For each individual entity we report: in bold the English version of the stimulus; in brackets, if the name required a translation to Italian for the experiment, the Italian words actually used, as well as the familiarity score (FAM) collected during the norming experiment. We use the same structure for each fine-grained and coarse-grained category, with the addition of average word lengths (LEN). Both familiarity and word length scores reported are averaged across individuals within the category.

People *(FAM=3.94, LEN=12)*

**Actor** *('attore', FAM=4.00, LEN=14)*	**Musician** *('musicista', FAM=3.80, LEN=9)*	**Politician** *('politico', FAM=4.09, LEN=13)*	**Writer**, *('scrittore', FAM=3.88, LEN=13)*
**Scarlett Johansson** *(FAM=4.06)*	**Madonna** *(FAM=3.69)*	**Silvio Berlusconi** *(FAM=4.45)*	**Ernest Hemingway** *(FAM=3.84)*
**Marylin Monroe** *(FAM=4.01)*	**Bob Dylan** *(FAM=3.90)*	**Hillary Clinton** *(FAM=3.87)*	**J.K. Rowling** *(FAM=3.87)*
**Woody Allen** *(FAM=3.87)*	**Freddie Mercury** *(FAM=3.96)*	**Barack Obama** *(FAM=4.21)*	**Sigmund Freud** *(FAM=4.03)*
**Quentin Tarantino** *(FAM=4.09)*	**W. A. Mozart** *(FAM=3.66)*	**Vladimir Putin** *(FAM=3.84)*	**William Shakespeare** *(FAM=3.78)*

**Places** *(FAM=3.80, LEN=9)*			
**City** *('città', FAM=4.11, LEN=6)*	**Country** *('paese', FAM=3.97, LEN=7)*	**Body of water** *('corso d'acqua', FAM=3.59, LEN=12))*	**Monument** *('monumento', FAM=3.52, LEN=11))*
**New York** *(FAM=4.09)*	**Spain** *('Spagna', FAM=4.09)*	**Mediterranean Sea** *('Mar Mediterraneo', FAM=4.30)*	**Pisa Tower** *('Torre di Pisa', FAM=3.78)*
**Amsterdam** *(fam=3.72)*	**Israel** *('Israele', FAM=3.93)*	**Atlantic Ocean** *('Oceano Atlantico', FAM=3.48)*	**Stonehenge** *(FAM=3.60)*
**Rome** *('Roma', FAM=4.51)*	**Germany** *('Germania', FAM=3.93)*	**River Nile** *('Nilo', FAM=3.33)*	**Sagrada Familia** *(FAM=3.42)*
**Paris** *('Parigi', FAM=4.12)*	**United Kingdom** *('Regno Unito', FAM=3.93)*	**Pacific Ocean** *('Oceano Pacifico', FAM=3.27)*	**Machu Picchu** *(FAM=3.27)*
